# ZnJ6 Is a Thylakoid Membrane DnaJ-Like Chaperone with Oxidizing Activity in *Chlamydomonas reinhardtii*

**DOI:** 10.3390/ijms22031136

**Published:** 2021-01-24

**Authors:** Richa Amiya, Michal Shapira

**Affiliations:** Department of Life Sciences, Ben-Gurion University of the Negev, Beer Sheva POB 653, Israel; amiyar@post.bgu.ac.il

**Keywords:** DnaJ-like chaperone, zinc finger domain (ZF), *Chlamydomonas reinhardtii*, redox, chloroplast

## Abstract

Assembly of photosynthetic complexes is sensitive to changing light intensities, drought and pathogens, each of which induces a redox imbalance that requires the assistance of specific chaperones to maintain protein structure. Here we report a thylakoid membrane-associated DnaJ-like protein, ZnJ6 (Cre06.g251716.t1.2), in *Chlamydomonas reinhardtii*. The protein has four CXXCX(G)X(G) motifs that form two zinc fingers (ZFs). Site-directed mutagenesis (Cys > Ser) eliminates the ability to bind zinc. An intact ZF is required for ZnJ6 stability at elevated temperatures. Chaperone assays with recombinant ZnJ6 indicate that it has holding and oxidative activities. ZnJ6 is unable to reduce the disulfide bonds of insulin but prevents its aggregation in a reducing environment. It also assists in the reactivation of reduced denatured RNaseA, possibly by its oxidizing activity. ZnJ6 pull-down assays revealed interactions with oxidoreductases, photosynthetic proteins and proteases. In vivo experiments with a *C. reinhardtii* insertional mutant (∆ZnJ6) indicate enhanced tolerance to oxidative stress but increased sensitivity to heat and reducing conditions. Moreover, ∆ZnJ6 has reduced photosynthetic efficiency shown by the Chlorophyll fluorescence transient. Taken together, we identify a role for this thylakoid-associated DnaJ-like oxidizing chaperone that assists in the prevention of protein misfolding and aggregation, thus contributing to stress endurance, redox maintenance and photosynthetic balance.

## 1. Introduction

Photosynthetic organisms are often challenged by biotic and abiotic stresses resulting in a redox imbalance that must be counteracted by the organism to survive. The redox status of chloroplast proteins is mainly controlled and influenced by photosynthetic light reactions, which can lead to an increased generation of reactive oxygen species (ROS) during stress [1,2]. Generation of ROS can occur in response to an imbalance between the excess of excited-state electron populations and the electron carriers. As a result, the excited electrons cannot be dissipated by their electron carriers, eventually leading to the generation of ROS and oxidative damage [2,3]. Proteins with cysteine residues and disulfide bridges are sensitive to redox changes that affect the redox status of these bridges leading to structural changes, impaired ability to function and to interact with partner proteins. Thus, the redox status of proteins plays an essential role in cell signaling and antioxidizing defense [4,5].

Here we describe a novel DnaJ-like protein, ZnJ6 (Cre06.g251716.t1.2), from *C. reinhardtii*. This protein has four cysteine-rich CXXCX(G)X(G) motifs that form two C4-type zinc fingers (ZFs) that can bind two zinc atoms [6] similar to those of DnaJ and can be categorized as a DnaJ-like protein (DnaJE1). DnaJE1 proteins lack all the other motifs of DnaJ, such as the J and G/F domains, and show no C-terminal homology to DnaJ [7]. The proteins of this group act independently of DnaK by direct substrate interaction and stabilization [8]. Furthermore, they do not require ATP fueling to assist in the prevention of aggregation as they bind their substrates directly. This was previously shown for ZnJ2 [9]. To date, 20 proteins of this family have been identified in *Arabidopsis* [7], but their orthologs in *Chlamydomonas* have not yet been determined, mainly due to their limited sequence similarities. Many DnaJ-like proteins function as chaperones and in the assembly of photosynthetic complexes. One example is the Bundle Sheath Defective Gene2 (BSD2) initially identified in maize, which is required for RuBisCO biogenesis [10,11]. Other examples are the thylakoid-associated DnaJ-like PSA2 and LQY1 proteins, which interact with components of the PSI [12] and PSII [13] complexes, respectively. Many DnaJEs reside in the chloroplast and have a chloroplast associated function [7,10,12,13].

Here we report that ZnJ6 is found in the chloroplast thylakoid membranes and explore its potential function as a chaperone and its response to changes in the redox environment. The localization of ZnJ6 was confirmed by biochemical subfractionation, followed by western analysis of different fractions using the organelle-specific protein markers. In addition to substrate-binding, we show that the ZF domain is required for protein stability at elevated temperatures. We analyzed the ZnJ6 response to the changing redox environment in the chloroplast using a ZnJ6 insertional mutant with reduced expression. Under heat and reductive stress, the WT strain grew better, indicating that ZnJ6 assists in resistance to these conditions. However, under oxidizing conditions, the mutant grew better than its background WT strain, compatible with our observation that ZnJ6 possesses an oxidizing activity. Furthermore, we show that under normal conditions, ZnJ6 is required for maintaining photosynthetic efficiency.

## 2. Results

### 2.1. ZnJ6 from C. reinhardtii Localizes to the Thylakoid Membrane of the Chloroplast

**Figure 1:**
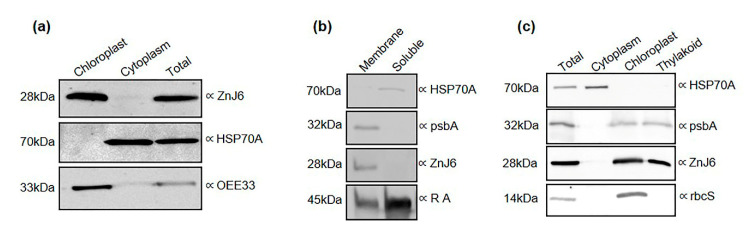
ZnJ6 is localized in the thylakoid membrane of the chloroplast. *C. reinhardtii* cells were grown until the late log phase and disrupted by nitrogen cavitation, and further subfractionated. (**a**) The purified chloroplasts, cytoplasm, and total proteins were subjected to western analysis using antibodies against oxygen-evolving enzyme (OEE33) as a chloroplast marker and HSP70A as a cytoplasmic marker; (**b**) The membrane and soluble fractions of *C. reinhardtii* cells were separated, and the presence of ZnJ6 in the membrane fraction was confirmed. Antibody against psbA served as a membrane marker, HSP70A for the soluble protein fraction, and Rubisco Activase (RA) is a marker for proteins in both the membrane and soluble fractions; (**c**) Thylakoid membranes were isolated using a sucrose step gradient. Samples taken from the total extracts, cytoplasm, chloroplast, and thylakoid fractions were subjected to western analysis using antibodies against psbA as a thylakoid marker, rbcS as a chloroplast marker, and HSP70A as a cytoplasmic marker. ZnJ6-specific antibodies indicated their presence in the thylakoid membrane. Equal protein loads for each fraction were determined by the bicinchoninic acid (BCA) protein assay (Ponceau staining is shown in the original blots in the supplemental data).

Cellular localization can serve as a preliminary indication of protein function [14]. We, therefore, determined the intracellular localization of ZnJ6 by biochemical subfractionation that separated the chloroplasts with their thylakoid membranes from the rest of the cell. The isolation of chloroplasts was confirmed by monitoring the ratio of OD_645_ and OD_663_. The isolated subcellular fractions, along with total cell protein, were validated by western analysis using antibodies against marker proteins typical for each fraction [15], anti-HSP70A for the soluble cytoplasmic fraction and antibodies against the oxygen-evolving enzyme (OEE33) and rbcS for the chloroplast fraction (Figure 1a). Resolving the membrane and soluble fractions of *C. reinhardtii* cells verified that the chloroplast membranes were included in the membrane fraction using antibodies against psbA. Subsequently, the thylakoid fraction was purified and verified by its interaction with the anti-psbA antibodies. The soluble fraction was verified by antibodies against HSP70A. Antibodies against Rubisco Activase (RA) showed that this protein was distributed unevenly between the membrane and soluble fractions with more protein in the soluble fraction (Figure 1b). The presence of ZnJ6 in the chloroplast membrane fraction was confirmed using specific antibodies that we raised (Figure 1a and Figure S1). We identified a transmembrane domain in ZnJ6 using the Phobius and TMHMM server (Figure S2) [16] and a chloroplast transit peptide using the ChloroP and TargetP servers (Figure S3). In conclusion, ZnJ6 is a membrane-associated protein present in the thylakoid fraction, as verified by specific antibodies (Figure 1b,c).

### 2.2. CD Analysis of Affinity Purified ZnJ6 Verifies that the Recombinant Protein Is Folded

**Figure 2:**
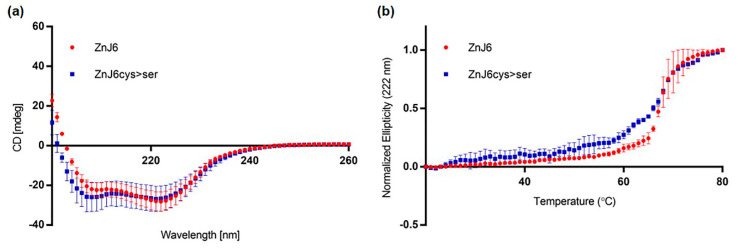
Recombinant ZnJ6 and ZnJ6cys > Ser-mutants are folded and stable up to 65 °C. (**a**) Circular dichroism (CD) single accumulation spectra (ellipticity represented in mdeg), ranging between 200 and 260 nm, were recorded for the ZnJ6 and the ZnJ6cys > Ser-mutant at RT to verify that the recombinant proteins are folded; (**b**) The single wavelength melting curve was generated at a constant wavelength of 222 nm at a temperature range of 20 to 80 °C. Normalized CD melting curves of ZnJ6 and Cys-mutant with relative values between 1.0 and 0.0 provide an overall comparative indication of the thermal stability of ZnJ6 and its ZnJ6cys > Ser-mutant. The WT recombinant ZnJ6 shows a more coordinated structure at elevated temperatures, as indicated by the steep slope. However, both proteins have the same midpoint of the melting curve at 65 °C. The figure shows the mean values obtained in three independent biological repeats; error bars show standard deviations.

**Figure 3:**
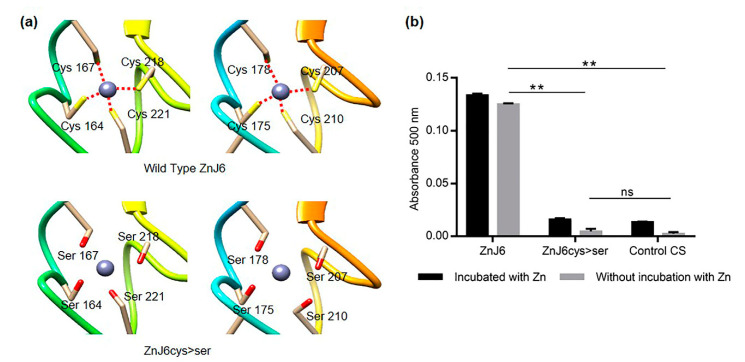
The cysteine-rich motif of ZnJ6 is required for its zinc-binding activity. (**a**) The structure of the Zinc-binding motif (Wild Type ZnJ6 and ZnJ6cys > Ser-mutant) was predicted using homology modeling (constructed by UCSF Chimera). The predicted loss of coordination (red dotted lines) with zinc due to the replacement of cysteine with serine in the ZnJ6cys > Ser-mutant; (**b**) Zinc-binding activity of ZnJ6 (WT and ZnJ6cys > Ser-mutant) was measured using 5 µM of purified recombinant proteins, either pre-incubated with 40 µM ZnCl_2_ to completely saturate the protein with Zn, or without pre-incubation with Zn, representing the Zn-binding status of the protein extracted from bacteria. The release of zinc was obtained using para-chloromercuribenzoic acid (PCMB), and the PAR–Zn^+2^ complex that was formed was monitored at 500 nm. Citrate synthase (CS) was used as a negative control. The Zn-binding assays were performed in triplicates. One-way ANOVA with Tukey’s multiple comparison test was used to evaluate the differences in Zn-binding; error bars show the standard deviations (**: *p* value ≤ 0.01, ns: not significant). A significant change was observed between the Zn-binding of the WT protein as compared to the ZnJ6cys > Ser-mutant or the CS control. There was no significant difference between samples with or without pre-incubation with ZnCl.

To examine whether the protein is folded, stable at high temperatures and to test whether the Zn-binding domain affects folding, we measured the circular dichroism (CD) spectra of recombinant WT and Cys-mutant (ZnJ6cys > Ser) proteins in which all eight cysteine residues in the predicted Zn-binding center were exchanged with serines (Figure 2a,b and Figure S4). Our structural model indicates that cysteine residues at positions 164, 167, 218, 221 could bind one zinc atom, and those at positions 175, 178, 207 and 210 could bind a second (Figure 3a). The measurements were taken with a single accumulation spectrum ranging between 200 and 260 nm at room temperature (RT) (Figure 2a) and at a constant wavelength of 222 nm with a temperature range from 20 °C to 80 °C, to examine whether elevated temperatures affect ZnJ6 structure (Figure 2b). The results indicate that although both the WT and ZnJ6cys > Ser were folded at RT (Figure 2a), they exhibited differences in their stability at higher temperatures, depending on the presence or absence of the ZF motif (Figure 2b). The melting curves show that both proteins remained folded up to 65 °C (midpoint of transition state). The slope of transition between the folded and unfolded states was gradual for the ZnJ6cys > Ser, unlike the WT protein. This difference indicates reduced cooperative interactions in the mutant compared to the WT protein (Figure 2b). The secondary structure of both proteins remained largely unaffected at RT regardless of the mutation in the ZFs.

### 2.3. Recombinant ZnJ6 Binds Zinc through Its Cysteine-Rich Motif

We evaluated zinc-binding by the Cys-rich domain using the Zn-binding assay with 4-(2-pyridylazo) resorcinol (PAR) and *p*-chloro-mercurybenzoate (PCMB). The assay was done using 3 μM of purified recombinant proteins (ZnJ6 or ZnJ6cys > Ser). The addition of 30 µM PCMB causes the release of zinc atoms from the protein leading to the formation of a colored complex due to its interaction with PAR, which is measured by absorbance at 500 nm [17]. Recombinant ZnJ6 and its ZnJ6cys > Ser-mutant were analyzed for zinc release just after purification (no pre-incubation) and after incubation with ZnCl_2_ (with pre-incubation). Our results show that the ZF domain of the WT protein is required for binding zinc. Zn-binding by the mutant protein, in which all eight Cys residues of the two ZFs were replaced with serines, loses its ability to bind zinc (Figure 3a) as zinc release was clearly lower, much like in the negative control (citrate synthase, CS) that does not contain a ZF domain (Figure 3b). Zinc was observed in both cases (irrespective of pre-incubation) with no significant difference. Therefore, the Cys residues that form the ZFs are required for the binding of zinc.

### 2.4. Recombinant ZnJ6 and the ZnJ6cys > Ser-Mutant Function as Chaperones that Prevent Thermal Aggregation of Temperature-Sensitive Substrates

**Figure 4:**
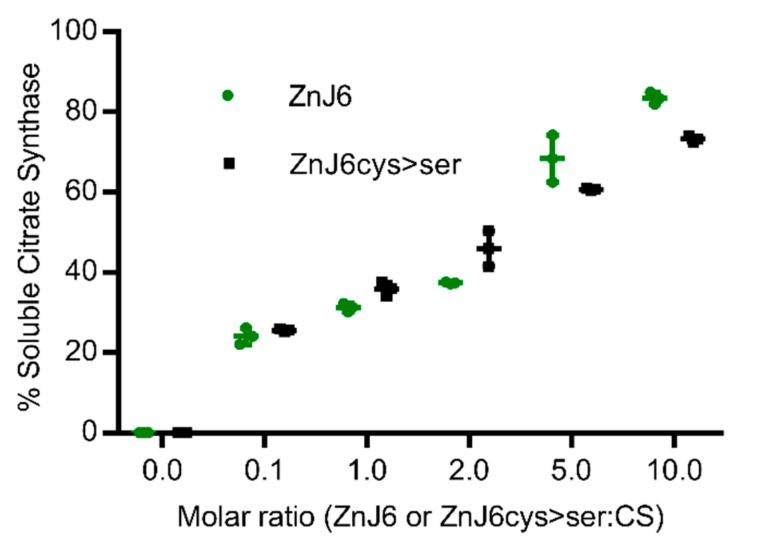
ZnJ6 and its ZnJ6cys > Ser-mutant have holding chaperone activity measured by the citrate synthase thermal aggregation assay. Citrate synthase (CS) was diluted into a refolding mixture at 42 °C (1 h) or into a refolding mixture with ZnJ6 (green circles). A parallel assay was performed with the ZnJ6cys > Ser-mutant (black squares). All assays were performed in the presence of increasing molar ratios relative to CS (ZnJ6: CS; 0:1, 0.1:1, 1:1, 2:1, 5:1, 10:1). The thermal aggregation of CS was measured by monitoring OD_360_ over time. The absorbance was used to calculate the percentage of soluble CS after 1 h of incubation at elevated temperatures. CS that was not subjected to increased temperature served as the 100% soluble control. CS alone incubated at 42 °C for 1 h served as the fully aggregated substrate. The *t*-test was used for statistical analysis, and the error bars show the standard deviation. This analysis showed that there is no significant difference between the holding chaperone activity of ZnJ6 and ZnJ6cys > Ser-mutant protein. The values show the mean of triplicate experiments.

To establish whether ZnJ6 can function as a chaperone, we performed the classical assay that measures the ability of a chaperone to prevent aggregation of a temperature-sensitive protein such as CS. This assay does not monitor refolding activities. CS is highly sensitive to temperature and loses its ability to fold already at 42 °C, as shown in our controls and by previous studies [18,19]. However, the CD measurements of ZnJ6 at increasing temperatures (Figure 2b) indicated that it remained folded up to 65 °C. The dose-dependent chaperone effect of ZnJ6 was determined by incubation in increasing molar ratios relative to the CS substrate (ZnJ6:CS were 0.1:1, 1:1, 2:1, 5:1, 10:1), at 42 °C for 1 h. CS aggregation was measured by monitoring OD_360_ over time as aggregation leads to increased absorbance. The results indicate that ZnJ6 could function as a chaperone and prevent substrate aggregation: in the presence of ZnJ6, CS exposed to 42 °C remained soluble even after 1 h, starting at a ratio of 1:1 and reaching maximum protection of 86% when mixed with ZnJ6 at a 10:1 (ZnJ6:CS) ratio indicating that the protective activity was dose-dependent (Figure 4). No significant difference was found between the chaperone activity of ZnJ6cys > Ser-mutant and WT ZnJ6 proteins, indicating that the ZnJ6 chaperone activity in preventing CS aggregation is independent of its ZFs.

A similar experiment used RA, an interacting partner of ZnJ6 that we identified by LC/MS-MS analysis (Table S1). Unfolding/aggregation of RA occurs already at 37 °C [20]. However, we found that the addition of ZnJ6 at a molar ratio of 3:1 (ZnJ6:RA) prevented the thermal aggregation of RA at 42 °C (Figures S5 and S6). These results further verify the chaperone role of ZnJ6 and are in accordance with previous findings highlighting the temperature sensitivity of RA [20].

### 2.5. ZnJ6 Is Unable to Reduce the Disulfide Bonds of Insulin but Prevents Its Aggregation in a Reducing Environment

**Figure 5:**
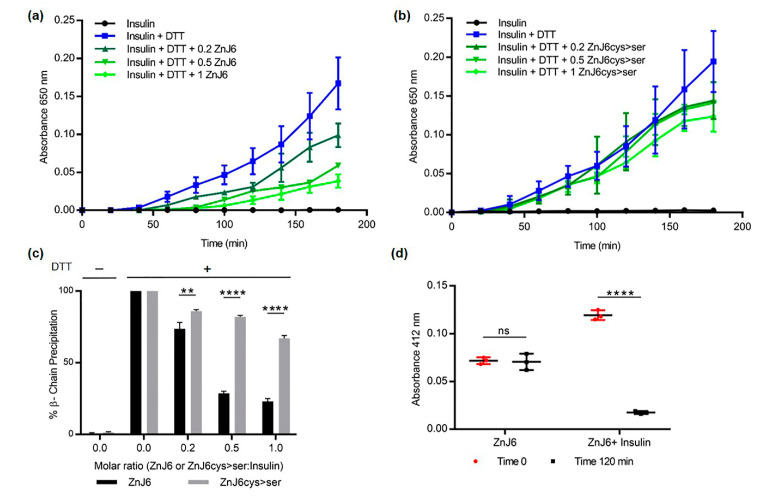
The C4-type zinc finger domain (ZF) is required to prevent aggregation of the reduced insulin chain. The insulin turbidity assay was used to examine the thiol-dependent activity of ZnJ6. Increasing molar ratios of ZnJ6 relative to insulin were added (0:1, 0.2:1, 0.5:1, 1:1), and precipitation of the insulin β-chain was measured at 650 nm for 3 h at 25 °C. A reaction containing insulin alone served as a control. (**a**) Precipitation was monitored in the presence of WT recombinant ZnJ6 and (**b**) ZnJ6cys > Ser-mutant. (**c**) Summary of end-point precipitation values obtained in the presence of different molar ratios of the ZnJ6 (black columns) and the ZnJ6cys > Ser-mutant (gray columns) after 3 h. (**d**) Protein-bound sulfhydryl (-SH) groups were calculated using the Ellman’s test before (red circles), and after incubation of ZnJ6 (black squares), with or without insulin, that was introduced at an equal molar ratio. Presented values are mean of triplicates, the Student’s *t*-test was used for statistical analysis, and the error bars are standard deviation (****: *p* value ≤ 0.0001, **: *p* value ≤ 0.01, ns: not significant). The results indicate that ZnJ6 significantly prevents the aggregation of Insulin chains as compared to the ZnJ6cys > Ser-mutant.

The thiol-dependent activity of ZnJ6 was examined in the insulin turbidity assay [21]. Insulin comprises two polypeptide chains, α and β, held together by disulfide bridges. The addition of a reducing agent such as dithiothreitol (DTT) causes these disulfide bridges to open, dissociating the chains. The reduced β-subunit aggregates and their precipitation can be measured at OD_650_ nm. In contrast to thioredoxins that accelerate precipitation of the insulin β-chain, ZnJ6 did not affect insulin chain release. However, when insulin was reduced in the presence of DTT, the addition of ZnJ6 in increasing molar concentrations (ZnJ6:insulin 0:1, 0.2:1, 0.5:1, and 1:1) prevented β-chain precipitation in a dose-dependent manner (Figure 5a). Moreover, in contrast to the WT protein, the ZnJ6cys > Ser-mutant failed to prevent precipitation of the insoluble reduced β-chain with the same efficiency (Figure 5b,c). Thus, although ZnJ6 lacked reducing activity, it could prevent insulin chain precipitation defining an essential role for the ZFs in preventing aggregation.

To further confirm the role of the cysteines in preventing the aggregation of insulin chains by ZnJ6, we monitored the amount of -SH groups with and without the insulin substrate, using 5-dithio-bis-(2-nitrobenzoic acid) (DTNB, Ellman’s reagent). DTNB binds to reduced -SH groups, forming a colored complex that can be measured by its absorbance at 412 nm. Our expectation was that insulin aggregation would not occur when the ZnJ6 -SH groups were occupied by the insulin -SH groups forming disulfide bridges and decreasing the total amount of -SH groups in the reaction mix. The amount of protein-bound -SH (PB-SH) groups in the mixture was determined before and after incubation in the presence of DTT (2 h) of recombinant ZnJ6 with and without insulin. The -SH groups of ZnJ6 remained unchanged after incubation with DTT. However, incubation with an equal molar ratio of insulin led to a dramatic decrease in the amount of ZnJ6-bound -SH groups supporting the formation of disulfide bridges between ZnJ6 and the reduced insulin chains (Figure 5d). In conclusion, ZnJ6 lacked any reducing activity by itself as it failed to precipitate the insulin chains in the absence of DTT. However, it did prevent the aggregation of reduced insulin chains by forming disulfide bridges between the Cys-rich motif of WT ZnJ6 and the reduced insulin chains.

### 2.6. ZnJ6 Promotes the Reactivation of Reduced and Denatured RNaseA by Its Oxidizing Activity

**Figure 6:**
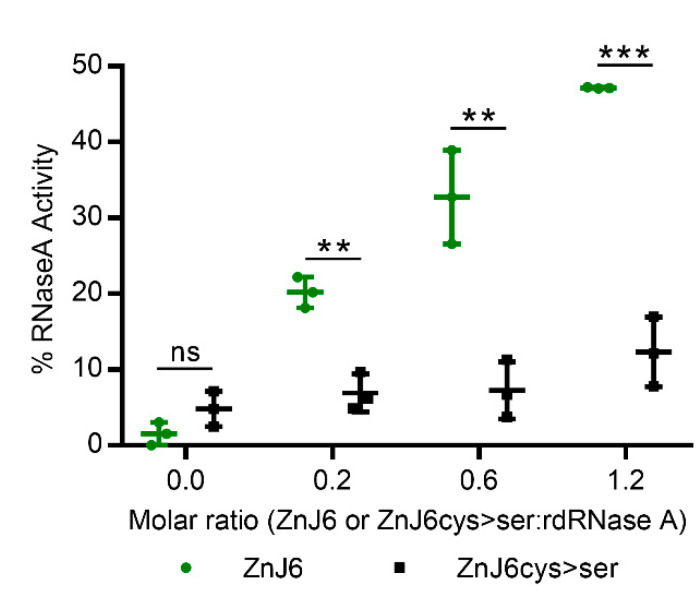
ZnJ6 affects the reactivation of reduced and denatured RNaseA. Reactivation of reduced and denatured RNaseA (rdRNaseA) generated using guanidinium hydrochloride (GnHCl) and dithiothreitol (DTT) was initiated 200-fold dilution into renaturation buffer to a final concentration of 25 μg/mL. Reactivation was conducted with and without ZnJ6, which was added at molar ratios of 0:1, 0.2:1, 0.6:1, and 1.2:1 relative to rdRNaseA. Reactivation was monitored in the presence of the WT ZnJ6 (green circles), or its ZnJ6cys > Ser-mutant (black squares). Aliquots were removed at various intervals and transferred into the assay mixture, and RNaseA activity was measured by monitoring the hydrolysis of cytidine 2′3′-cyclic monophosphate at 284 nm. The points represent the final percentage of RNaseA activity compared to the native RNaseA after 1 h of reactivation. Presented values are mean of triplicates, *t*-test was used for analysis, and the error bars show standard deviations (*** and **: *p* values ≤ 0. 001, and 0.01, respectively, ns: not significant).

To further investigate the thiol-oxidative capacity of ZnJ6, we measured the oxidative reactivation of reduced and denatured RNaseA (rdRNaseA) in the presence of increasing molar ratios of ZnJ6. The native structure of RNaseA is stabilized by four disulfide bridges. Once these bonds are reduced and the protein is denatured by guanidinium hydrochloride (GnHCl), it loses its enzymatic activity. Recovery of rdRNaseA activity requires the native disulfide bonds to reform, thus stabilizing the refolded structure. Upon removal of the GnHCl denaturant, the rdRNaseA alone failed to regain its activity by spontaneous refolding. However, activity was restored by the addition of ZnJ6 in increasing molar ratios to rdRNaseA (ZnJ6:rdRNaseA, 0:1; 0.2:1; 0.6:1; and 1.2:1). Within one h following removal of the denaturant, rdRNaseA activity was restored in a dose-dependent manner from 20% for the 0.2:1 molar ratio to 50% at the 1.2:1 molar ratio (Figure 6). Similar molar ratios of the ZnJ6cys > Ser-mutant showed only a minimal effect and did not refold the RNaseA with the same efficiency. Thus, ZnJ6 could assist the reformation of native disulfide bridges in the reduced and denatured RNaseA in a ZF-dependent manner restoring its activity.

### 2.7. ZnJ6 Interacts with Photosynthetic Proteins

The protein interactome plays an important role in predicting the function of target proteins. We, therefore, performed ZnJ6 pull-down assays to identify interacting partners (Figure S7). In vivo overexpression of ZnJ6 tagged with the streptavidin-binding peptide (SBP)tag did not achieve a level that could support efficient affinity purification. We, therefore, used a recombinant protein that was expressed as a fusion with two tags, the maltose-binding protein (MBP) and the streptavidin-binding peptide (SBP). Following purification over an amylose column, the MBP tag was cleaved off and removed by affinity purification of the recombinant protein over a streptavidin–Sepharose column. The purified SBP-ZnJ6 protein was dialyzed, incubated with solubilized chloroplast extracts for 2 h and allowed to bind to the streptavidin–Sepharose column along with its interacting partners. Similarly, treated recombinant SBP-tagged maltose-binding protein (MBP) served as an experimental control. The protein along with its interacting proteins were eluted using biotin; eluted fractions were analyzed by mass spectrometry (MS) using the MaxQuant software. Protein identification was set at less than a 1% false discovery rate. Label-free quantification (LFQ) intensities were compared for the three SBP-ZnJ6 biological repeats and the three MBP-SBP controls with the Perseus software platform using the Student’s *t*-test analysis.

**Figure 7:**
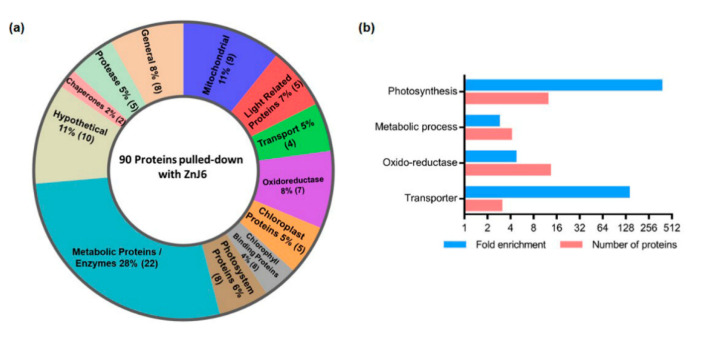
Protein categories associated with ZnJ6 in pull-down assays. The proteins pulled down with ZnJ6 were determined by LC–MS/MS analysis in triplicate and compared to control pull-down assays performed with a non-related maltose-binding protein (MBP) protein that was treated similarly. The proteins were identified by the MaxQuant software using Phytozome database annotations. Differences between the proteomic contents of the ZnJ6- and MBP-pulled-down fractions were determined using the Perseus statistical tool. Proteins with eight-fold enrichment compared to the control, with *p* < 0.05, were categorized. (**a**) Manual categorization of proteins into functional groups along with their relative abundance represented by the respective area (%) and the number of proteins (in brackets). The hypothetical group contains proteins with non-defined functions; (**b**) BLAST2GO enrichment was determined by the biological process with a minimum threshold of two-fold enrichment for the associated proteins compared to their gene abundance in the genome data set.

The enrichment threshold (LFQ intensities of SBP-ZnJ6 subtracted from the MBP-SBP controls) was set to a log2-fold change ≤ −3 (8-fold enrichment compared to control) with *p* < 0.05. The filtered proteins were categorized into functional groups both manually and by BLAST2GO based on enrichment for biological processes. The manual categorization indicated that the ZnJ6 interacting proteins comprised photosynthetic PSI components, transporters, ubiquinols, chaperones, and chlorophyll-binding proteins (Figure 7a,b and Table S1). Notably, ZnJ6 did not interact with the highly abundant subunits of RuBisCO (LS/SS), supporting the specificity of binding between ZnJ6 and its associated proteins. A parallel classification was done using BLAST2GO enrichment analysis, setting a minimum threshold of two-fold enrichment for the associated proteins compared to their gene abundance in the genome data set. Using this approach, we also observed high enrichment of photosynthetic proteins and transporters in the ZnJ6 interactome, along with proteins related to oxidoreductases and metabolism. The association with proteins of photosynthetic complexes suggests that ZnJ6 could have a specific role in the assembly of these photosynthetic complexes.

### 2.8. Chlamydomonas Mutant Cells Expressing a Low Level of ZnJ6 (*∆*ZnJ6) Show Higher Tolerance to Oxidative Stress, but Are Sensitive to Reducing Stress

As ZnJ6 is a redox-regulated chaperone, we examined its effects on the cells in changing redox environments. For this purpose, we examined a *Chlamydomonas* insertional mutant expressing a low level of ZnJ6 (CLiP mutant, LMJ.RY0402.048147) denoted ∆ZnJ6. The ∆ZnJ6 mutant was first confirmed using colony PCR and western analysis with anti-ZnJ6 antibodies (Figure S8). The background strain, cc-4533, served as the WT control strain for all the in vivo experiments.

**Figure 8:**
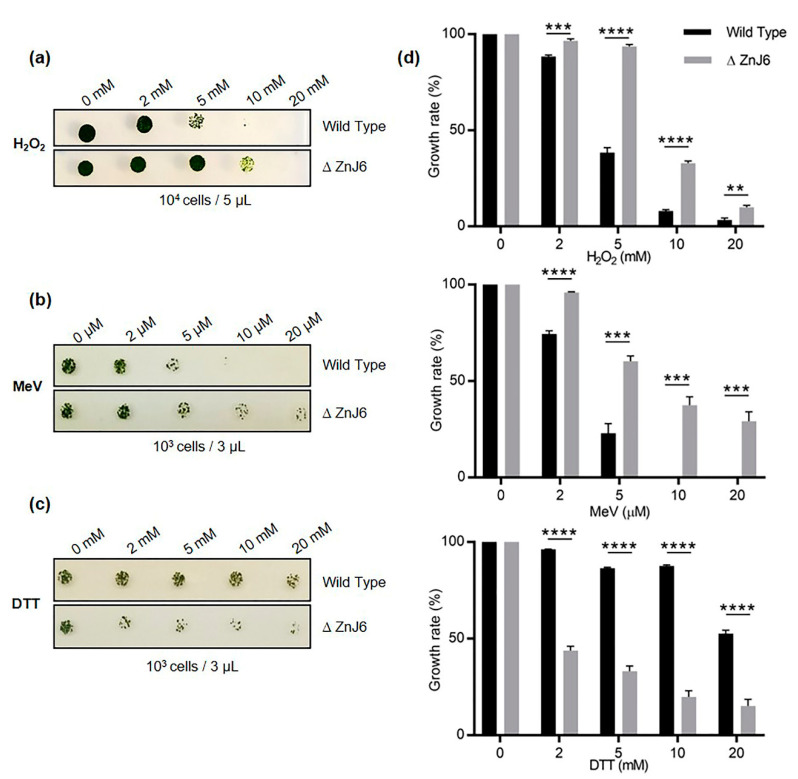
ZnJ6 knock-down mutant of *C. reinhardtii* (∆ZnJ6) shows tolerance to oxidative stress but increased sensitivity to reducing conditions. (**a**), (**b**) WT and ∆ZnJ6 cells were exposed to oxidizing conditions by incubation with increasing concentrations of hydrogen peroxide (H_2_O_2_) and methyl viologen (MeV) for 1 h respectively. The cells were washed, and growth was monitored on high salt (HS) plates. (**c**) Cells were exposed to reducing conditions by incubation with DTT for 2 h. The cells were washed, and growth was monitored by plating over HS plates. (**d**) Growth variations of cells treated with H_2_O_2_, MeV, or DTT, as shown in panels A–C, were measured using the MultiGauge software. The growth of untreated cells served as control (100%). The figure is representative of one of the three repeats. *t*-test was used for analysis, and the error bars are standard deviations. (****, ***, **: *p* values≤ 0.0001, 0.001 and 0.01, respectively).

To examine the effect of this mutation on growth and its response to different oxidizing environments, cells were grown to mid-log phase in high salt (HS) medium with a light intensity of 150 µmol/s/m^2^ in the presence of paromomycin for selective maintenance of the mutation. The cells were exposed to different concentrations of hydrogen peroxide (H_2_O_2_) (0, 2, 5, 10, and 20 mM) and methyl viologen (MeV) (0, 2, 5, 10, and 20 µM) for one h. The oxidizing agents were then removed by washes. Under increased oxidizing conditions, the mutants were more tolerant to oxidative stress than the WT cells (Figure 8a,b).

However, the opposite effect was observed when their growth was compared under reducing conditions, i.e., when cells were treated with DTT (0, 2, 5, 10, and 20 mM) for 2 h followed by washes. Cells were then spotted and allowed to grow for 5 days at 23 °C on HS plates. Whereas WT cells grew well at all DTT concentrations, the growth of the ∆ZnJ6 mutant cells was severely compromised (Figure 8c). DTT can easily cross membranes and cause disulfide bond reduction leading to reductive stress. Growth under reducing conditions can lead to changes in protein structure, resulting in their toxic aggregation [22]. Since the growth of the ΔZnJ6 mutant was impaired by DTT-treatment compared to WT cells, we hypothesize that the oxidizing activity of ZnJ6 is involved in counteracting the reducing activity of DTT in vivo, thereby making the WT more resistant to DTT. This result corroborates our finding that ZnJ6 could prevent the aggregation of reduced insulin chains in vitro.

### 2.9. The ZnJ6 Insertional Mutant (*∆*ZnJ6) Is Sensitive to Heat Stress

**Figure 9:**
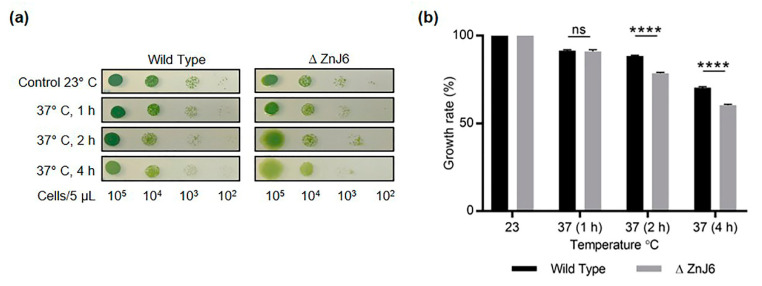
The ∆ZnJ6 mutant is sensitive to heat stress. (**a**) *Chlamydomonas* cells (WT and ∆ZnJ6) grown to mid-log phase in HS medium were serially diluted. Aliquots (5 μL) from each dilution were spotted on HS plates and incubated at 37 °C for 1, 2 and 4 h. Control plates were maintained at 23 °C. All the plates were then allowed to grow for 5 days under continuous illumination at 23 °C. Decreased growth and an increase in yellowing are observed in the ∆ZnJ6 cells that were incubated at 37 °C for at least 2 h; (**b**) Densitometric analysis of the growth observed in panels a (taken from cells spotted at a dilution of 10^3^ cells per 5 µL) using myImageAnalysis (Thermo Fisher Scientific) software. Growth at 23 °C was considered as 100%. The figure represents one of three repeats. The Student’s *t*-test was used to analyze the difference in growth between WT and ∆ZnJ6; error bars show the standard deviation values (****: *p* value ≤ 0.0001, ns: not significant).

To further elaborate on the role of ZnJ6 under stress conditions, we evaluated the response of the ∆ZnJ6 mutant to heat stress. We spotted 5 µL of the ∆ZnJ6 and WT cells (with concentrations that ranged between 10^5^ to 10^2^ cells/5 µL) on HS plates and incubated them at 37 °C for different times (0, 1, 2, 4 h). Following this treatment, the plates were grown at 23 °C for five days. Figure 9a shows that exposure to an elevated temperature for 2 h or more resulted in chlorophyll reduction in the ∆ZnJ6 mutant, with the cells becoming yellow. The growth of the mutants was slower than that observed for the WT control (Figure 9a).

Image analysis using myImageAnalysis software (Thermo Fisher Scientific) was used for densitometric analysis. The growth of both WT and ∆ZnJ6 cells was reduced in response to the heat stress compared to their growth at 23 °C; however, the mutant growth was significantly impaired (Figure 9b). Thus, ZnJ6 may assist the cells to withstand heat stress and to acclimatize to changing temperatures by preventing the irreversible aggregation of temperature-sensitive proteins such as RA and allowing continued growth.

### 2.10. ZnJ6 Activity Is Required for Photosynthesis under Optimal Growth Conditions

**Figure 10:**
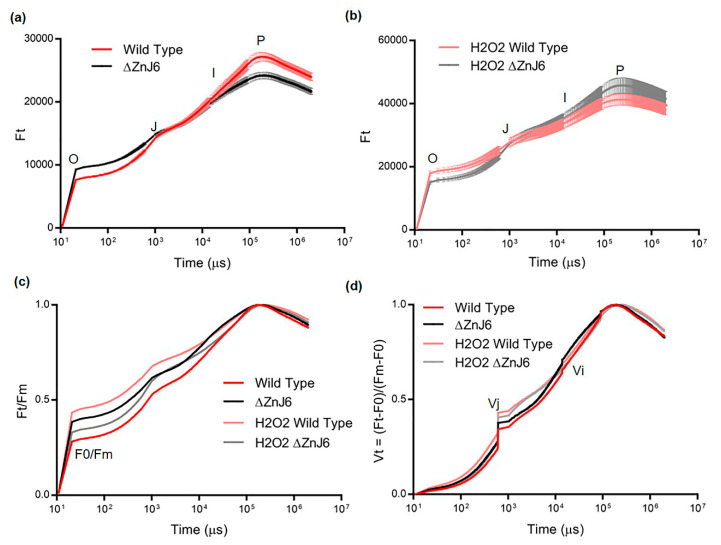
Differences in the photosynthetic efficiency of the Wild Type (WT) and ∆ZnJ6 mutant under optimal and oxidizing conditions. (**a**) The originally measured chlorophyll a fluorescence induction kinetics (OJIP transient) of the WT and ∆ZnJ6 *Chlamydomonas* cells under optimal environment, showing the polyphasic rise with minimum level fluorescence (O, corresponds to F0) and maximum (P, corresponds to Fm). The curve represents the mean of three independent experimental repeats, error bars represent standard deviations. Red and black curves represent WT and ΔZnJ6 values under normal conditions, respectively. (**b**) OJIP transient of WT and ∆ZnJ6 *Chlamydomonas* cells under oxidizing conditions (2 mM H_2_O_2_ for 1 h) also showing the polyphasic rise. The curve represents the mean of three independent experimental repeats, error bars represent standard deviations. Pink and gray curves represent WT and ΔZnJ6 values under oxidizing conditions, respectively. (**c**) Ft/Fm values of WT and ∆ZnJ6 cells under optimal and oxidizing conditions. Curve colors are described in a and b. (**d**) The relative variable fluorescence (Vt) of WT and ∆ZnJ6 *Chlamydomonas* cells under optimal and oxidizing conditions to compare the transient dynamics of the OJIP curve obtained for the WT and ZnJ6 cells. The curve was generated from the mean values obtained from three independent experimental repeats. Curve colors are described in **a** and **b**. Ft = chlorophyll fluorescence at time t. F0 = fluorescence intensity at 50 µs (O). It is the minimum fluorescence from dark-adapted cells. Fm = maximal fluorescence intensity at peak P. Vt = (Ft − F0)/(Fm − F0) represents values that were double normalized to Fm and F0. Vj and Vi = relative variable fluorescence at the intermediate J and I step, respectively.

As ZnJ6 binds strongly to photosynthetic proteins (Figure 7 and Table S1), we examined its role in photosynthesis under oxidizing and normal conditions. The OJIP curve is a powerful tool to investigate photosynthetic efficiency by measuring a minute fraction of absorbed photons that escape as chlorophyll fluorescence and form a typical curve. Any stress, damage, mutation to the photosynthetic machinery influences the shape of the curve that shows the origin (O), the (J) and (I) intermediate points and the fluorescence peak (P). Different stress conditions such as high-temperature, salinity and drought can lead to an increase in F0 (fluorescence at point O) and a decrease in Fm (fluorescence at point P) [23]. The rise in F0 could result from the dissociation of the light-harvesting complex (LHCII) from the PSII core complexes [23,24,25]. However, this could not explain the reduction in Fm values. This reduction was proposed to be related to the denaturation of proteins in the photosynthetic machinery (chlorophyll proteins) [23,26]. Our results (Figure 10a) show high F0 and low Fm values for the ΔZnJ6 mutant in an optimal environment as compared to the control WT background strain. The increased sensitivity of proteins of the photosynthetic machinery in the ΔZnJ6 mutant could explain the reduced Fm values that were observed under optimal conditions. We also consider the absence of the chaperone activity of ZnJ6 as a potential explanation for the increase in F0 and decrease in Fm values in the ΔZnJ6 mutant under normal conditions. Furthermore, in the absence of ZnJ6, the repair of PSII components could be less efficient, leading to the observed changes in the OJIP curves and possibly also affecting PSI components. Our pull-down assays show that the recombinant ZnJ6 protein interacts with chlorophyll a- and b-binding proteins, as well as with proteins of PSI (Table S1 and Figure 10a). Hence, due to its chaperone activity, ZnJ6 could be involved in stabilizing proteins of the photosynthetic machinery and maintaining the electron flow between PSII and PSI. Therefore, its absence resulted in increased F0 and reduced Fm in the ΔZnJ6 mutant. Thus, the photosynthetic activity of the ΔZnJ6 mutant is reduced under normal conditions. However, a relative increase in F0 and a decrease in Fm were observed during oxidizing conditions for the WT control cells as compared to the ΔZnJ6 mutant (Figure 10b). This suggested an increased sensitivity of the WT cells to the oxidizing conditions. The observation is in accordance with our growth and survival analysis shown in Figure 8a,b, where the growth of the ΔZnJ6 mutant cells is improved under oxidizing conditions.

The increase in F0/Fm can be the consequence of reduced photosynthetic efficiency due to stress, as suggested by a previous report [27]. We show here a decreased F0/Fm ratio under normal conditions for the WT cells as compared to the ΔZnJ6 mutant (represented by Ft/Fm at origin O), showing the efficient photosynthetic activity of WT under optimal conditions. We further show increased F0/Fm ratios for WT as compared to ∆ZnJ6, following treatment with H_2_O_2_ (compare pink to gray curves in Figure 10c), indicating that the mutant has enhanced resistance to oxidizing conditions.

For comparison between the measurements of the different strains and conditions, the Vt curves (Vt represents the relative variable fluorescence) were generated by Ft−F0/Fm−F0 to perform a double normalization that eliminates the variability of the F0 and Fm values (Figure 10d). A previous report shows that the impairment in the light-harvesting complex results in reduced Fm−F0 values. These can eventually lead to an increase in Vt (Ft−F0/Fm−F0). Furthermore, the higher Vj values (Vt at point J) indicate a reduced plastoquinol pool [23,25,28]. We have observed that the ∆ZnJ6 mutant has slightly higher Vj values under optimal conditions (Figure 10d, compare black and red curves). Therefore, the presence of the ZnJ6 protein could help in maintaining the plastoquinol pool oxidized, improving photosynthetic efficiency. This difference was minimized at the Vi point. Under oxidizing conditions, there is an increase in the Vj values for both WT and ∆ZnJ6 as compared to normal conditions (compare pink to red and gray to black curves). However, the WT Vj is higher under oxidizing conditions (pink curve) as compared to the ΔZnJ6 mutant (gray curve), indicating the higher sensitivity of WT cells to oxidizing conditions.

## 3. Discussion

We report that the *C. reinhardtii* DnaJ-like protein ZnJ6 (Cre06.g251716) is associated with the chloroplast thylakoid membrane. We established this using biochemical fractionation assays and western analysis. These experimental results are supported by our primary sequence analysis indicating that ZnJ6 has both a putative chloroplast transit peptide and a transmembrane domain. ZnJ6 is a ZF oxidase that contains four cysteine-rich CXXCX(G)X(G) motifs that form two C4-type ZFs that bind two zinc atoms. These ZFs confer a stable, coordinated structure to the protein at elevated temperatures, as we show by circular dichroism (Figure 2a,b). However, ZnJ6 has distinct properties and cannot be considered to be the algal ortholog of the DnaJE chaperones of higher plants such as maize BSD2 (GRMZM2G062788_T01) [9] that is a chloroplast protein but lacks a transmembrane domain [29,30].

ZF domains are known to be involved in protein–protein interactions and can even contribute to the ability of chaperones to identify denatured substrate proteins [8]. We, therefore, explored the role of the cysteine-rich motif in chaperone activities of ZnJ6 using recombinant proteins (WT ZnJ6 and its ZnJ6cys > Ser-mutant) in classical in vitro chaperone assays. Based on the citrate synthase prevention of aggregation assay, we show that the chaperone “holding activity” of ZnJ6 does not require its Cys-rich domain. However, this domain is required for the redox activity of ZnJ6.

ZnJ6 activity varies from other redox-related chaperones. For example, whereas thioredoxin can induce precipitation of the insulin β-chain [21], ZnJ6 did not have this reducing activity. However, it could protect these chains from precipitation in the presence of a reducing agent such as DTT. This activity required the Cys-rich domain. We, therefore, conclude that ZnJ6 can not reduce disulfide bonds in its target proteins. Overall, this also excludes the possibility that ZnJ6 has protein disulfide isomerization activity (PDI). Based on the RNaseA assay, we show that ZnJ6 has oxidizing activity and assists in stabilizing the spontaneous native folding of rdRNaseA as we do not observe the restoration of activity in the absence of ZnJ6. This activity was dependent on the presence of a functional ZFs. Thus, ZnJ6 is a chaperone that can hold its target to prevent aggregation; it lacks reducing activity but can promote disulfide-bridge formation in its target proteins.

To further expand our general understanding of ZnJ6 protein interactions and in which context they occur, we performed a pull-down assay in which we affinity-purified chloroplast extracts of *Chlamydomonas* over immobilized recombinant ZnJ6. ZnJ6 co-purified with the majority of photosynthetic proteins (12), oxidoreductases (13), proteases (5), transporters (4) and chaperones (2), suggesting that it could be responsible for chaperoning a multitude of substrate proteins. However, at this stage, we cannot relate these findings to a direct interaction between these proteins and ZnJ6. The association of ZnJ6 with oxidoreductases could indicate its involvement in maintaining a subcellular redox balance. Co-purification of ZnJ6 with photosynthetic proteins (the majority of photosystem I proteins) could indicate a role in the assembly of the photosynthetic complex, as shown for other DnaJ-like proteins such as PSA2 [12] and LQY1 [13]. ZnJ6 localization in the thylakoid membranes could restrict its function to complexes that are formed in the membranes. ZnJ6 could also affect the biosynthesis of thylakoid membrane components as these are also coordinated with the photosynthetic machinery.

We have observed the enrichment of metabolic enzymes in the BLAST2GO analysis of ZnJ6-interacting proteins. This observation is in agreement with growing evidence for dual functions observed for metabolic enzymes, among them RNA-binding activities. Such activities could be related to their moonlighting activities, although other explanations are possible. We previously reported that RuBisCO LSU possesses RNA-binding activity, which is related to its regulation under oxidizing conditions [31]. The RNA-binding activity of metabolic enzymes was recently expanded to a multitude of enzymes [32], raising intriguing possibilities for such activity on the part of ZnJ6 [33].

To elaborate on the role of ZnJ6 in redox responses, we performed in vivo experiments monitoring the growth of *C. reinhardtii* ZnJ6 knock-down mutant cells that expressed a low level of ZnJ6 in different redox environments. These assays indicate that the ΔZnJ6 mutants are more tolerant to oxidative stress caused by short incubations with H_2_O_2_ or MeV compared to WT cc-4533 cells. The mechanism behind this activity is not fully resolved, but it could be related to the oxidizing features of ZnJ6. A similar physiological effect was reported for the FtsH5-interacting protein (FIP, At5g02160) in *Arabidopsis*. This protein has been found in mosses and higher plants and is another DnaJ-like protein that lacks the typical J-domains [34]. ZnJ6 and FIP have four and two cysteine-rich motifs, respectively; mutants of both proteins show tolerance to oxidative stress. ZnJ6 associates with an FtsH-like protease as well as with members of the ClpP protease complex. ClpP proteases are involved in the maintenance of chloroplast protein homeostasis [35,36]. Furthermore, chloroplast chaperones are known to regulate protease activities and function in synergy to maintain protein quality control [37]. Therefore, ZnJ6 could be involved in protein quality control and in regulating protein homeostasis.

In contrast to the improved growth of the ΔZnJ6 mutant cells under oxidizing conditions, these cells were more sensitive to a reducing force induced by DTT, showing impaired growth as compared to WT cells. DTT can easily cross the membrane and cause disulfide bond reduction, leading to reductive stress [22]. We assume that in the presence of DTT, protein structures of the chloroplast proteins could be affected by the reduction of disulfide bridges. Thus, the oxidizing activity of ZnJ6 could restore disulfide bridge formation, thus protecting protein structures in the WT cells. In the absence of ZnJ6, the reductive force of DTT could impair protein structures and possibly lead to their aggregation. Thus, ZnJ6 could provide resistance to the WT cells against DTT induced stress. This hypothesis is supported by the results of the in vitro insulin aggregation assay.

Shifting *Chlamydomonas* cells from 25 °C to 37 °C induces a heat stress response [38]. Our data suggest that ZnJ6 is involved in protection against heat stress since the exposure of the ΔZnJ6 mutant cells to elevated temperatures for 2–4 h resulted in the degradation of chlorophyll and impaired growth. Thus, the mutant cells appear to be much more sensitive to heat stress (37 °C) than WT cells. This could possibly be attributed to the chaperone activity of ZnJ6 under heat stress and is further supported by our finding that ZnJ6 interacts with ClpP proteases involved in the chloroplast unfolded protein response, UPR, and proteostatic processes [39]. The ability of ZnJ6 to protect a substrate protein from heat-induced aggregation was shown in vitro in the CS assays. ZnJ6 structure is stable up to 65 °C, which would enable such activity. ZnJ6 also interacts with the temperature-sensitive catalytic chaperone RA in our pull-down assays. Unfolding and aggregation of RA occur at 37 °C [20]. RA is a chloroplast protein responsible for RuBisCO activation and the primary cause of reduced photosynthesis at elevated temperatures due to its temperature sensitivity [40,41]. In our in vitro assays, we showed that ZnJ6 prevents the aggregation of RA at 42 °C. This could suggest a role for ZnJ6 in preventing the aggregation of temperature-sensitive proteins in the chloroplast, thus assisting the growth and survival of cells at elevated temperatures.

From the perspective of stress studies, the OJIP fluorescence transient measurements may serve as an indication of the physiological condition of plants [24]. The OJIP experiments using the ΔZnJ6 mutant and its WT control cells under optimal environmental conditions showed a higher F0 and lower Fm values for the ΔZnJ6 mutant (Figure 10a). This could indicate a lower photosynthetic activity of the ΔZnJ6 mutant in an optimal environment and could suggest that the ZnJ6 protein plays an important role in maintaining the functional state of the protein components in the photosynthetic machinery, as indicated elsewhere [23,26]. This is also supported by the observation that the recombinant ZnJ6 protein interacts with the Chlorophyll-binding proteins a and b along with PSI components in pull-down assays. An increase in F0/Fm (Figure 10c) and Vj values (Figure 10d) for the ΔZnJ6 cells under normal conditions also suggest that the ZnJ6 protein could help in maintaining the plastoquinol pool oxidized, thereby improving the photosynthetic efficiency of WT. However, under oxidizing conditions (2 mM H_2_O_2_), we observed that the ΔZnJ6 mutant displayed a better photosynthetic performance as compared to the WT cells (Figure 10b–d) indicating that in an oxidizing environment, ZnJ6 was not required as under normal conditions. The higher photosynthetic efficiency of WT cells under optimal conditions could also be explained by the oxidizing activity of ZnJ6 that enables the electron flow from PSII to PSI. This is also supported by the co-purification of PSI components with ZnJ6 in the pull-down assays (Figure 7 and Table S1). This association could also be explained by the putative involvement of ZnJ6 in the PSI assembly.

In conclusion, ZnJ6 is a DnaJ-like oxidizing chaperone that has no reducing power. It was shown to localize in the thylakoid membrane of *Chlamydomonas*. ZnJ6 increased the photosynthetic activity of WT cells under normal conditions, and its reduced expression in the ΔZnJ6 mutant decreased this activity. However, the ΔZnJ6 mutant cells were more tolerant to oxidizing conditions as compared to WT cells, indicating that the oxidizing activity of ZnJ6 was not required under oxidizing conditions and even increased the sensitivity of the WT cells to grow under these conditions. This is also supported by the results obtained under reducing conditions (DTT), where the ΔZnJ6 mutant growth was inferior to WT cells. ZnJ6 was also shown to be required for growth at elevated temperatures, in line with its ability to prevent protein aggregation at elevated temperatures using in vitro assays (Figure 4 and Figure S6). ZnJ6 also assists in maintaining a photosynthetic balance in an optimal environment as it interacts with PSI complexes and may contribute to the oxidative status of PSI electron acceptors. We also hypothesize that ZnJ6 could be involved in the assembly of photosynthetic complexes, as observed for other DnaJ-like proteins [12,13].

## 4. Materials and Methods

### 4.1. Isolation of RNA, cDNA Synthesis, and Cloning

Early log-phase cells of *Chlamydomonas reinhardtii* cc-125 were measured by taking absorbance at 750 nm and counting (OD_750_ = 0.25 − 0.35; 2 × 10^6^ cells/mL). The cells were used to isolate total RNA by the TRI Reagent (Sigma-Aldrich, Rehovot, Israel) protocol. The cDNA was synthesized using the high capacity cDNA reverse transcription protocol (Applied Biosystems, Foster City, CA, USA) with 1 µg RNA as a template. Bacterial clones for recombinant protein expression were generated as described in the Supporting methods S1, S2 and, S3 section and the primers used are listed in Tables S2 and S3.

### 4.2. Chlamydomonas Strains and Growth Conditions

*Chlamydomonas* strains ((cc-125 and cc-4533) were grown and maintained on Tris-acetate-phosphate (TAP) plates (1 L of TAP media contains 50 mM of 2-amino-2-(hydroxymethyl)-1,3-propanediol (Tris) base, 1 mL Hunter’s trace elements (50 gr EDTA, 22 gr ZnSO_4_∙7H_2_O, 11.4 gr H_3_BO_3_, 5.06 gr MnCl_2_ ∙4H_2_O, 4.99 FeSO_4_∙7H_2_O, 1.61 gr CoCl_2_∙6H_2_O, 1.57 gr CuSO_4_∙5H_2_O, 1.10 gr Mo_7_O_24_(NH_4_)_6_∙4H_2_O in 800 mL), 0.1% acetic acid, 1 mL concentrated phosphate buffer (10.8 gr K_2_HPO_4_, and 5.6 gr KH_2_PO4 in 100 mL ddH_2_O) and 25 mL TAP salt solution (16 gr NH_4_Cl, 4 gr MgSO_4_∙7H_2_O, 2 gr CaCl_2_∙2H_2_O in 1 L ddH_2_O) at 23 °C with light intensity of 100 µmol/s/m^2^. The knock-down ZnJ6 CLiP mutant LMJ.RY0402.048147 (∆ZnJ6) with insertion in the 3′ UTR along with the background strain cc-4533 was obtained from the *Chlamydomonas* Resource Center [42]. The knock-down mutant was maintained on 10 µg/mL paromomycin on TAP plates and verified by colony PCR followed by western analysis. A fresh colony was inoculated in 10 mL TAP media with required antibiotics followed by large-scale culturing in high salt (HS, 1 L of HS media contains 5 mL of concentrated Beijerink’s (100 gr NH_4_Cl, 4 gr MgSO_4_∙7H_2_O, 2 gr CaCl_2_∙2H_2_O in 1 L), 5 mL of concentrated phosphate buffer and 1 mL of Hunter’s trace metals) or TAP media (as per requirement), with 12 h dark/light cycles (at 100–150 µmol/s/m^2^) and constant rotary shaking at 100 rpm.

### 4.3. Recombinant Protein Purification

An overnight bacterial starter culture (10 mL) in LB medium supplemented with 100 μg/mL ampicillin and 25 μg/mL chloramphenicol (for Rosetta strain only) was inoculated into 1 L of LB supplemented with required antibiotics and 1% glucose. Expression of the SBP-tagged pMBP-GB1-ZnJ6 (see method S1) was induced by the addition of 0.2 mM IPTG when cells reached OD_600_ = 0.5–0.7, at 20 °C for 16 h. The culture was harvested and resuspended in lysis buffer (20 mM Tris-HCl, pH 7.4, 200 mM NaCl, 1 mM EDTA) containing 0.1% Brij 58 (Sigma-Aldrich, Rehovot, Israel), a protease inhibitor (PI) cocktail, and 5 μg/mL DNaseI. The cells were disrupted in a French press at 1500 PSI and centrifuged at 45,000 rpm (Beckman 70 Ti rotor). The supernatant was loaded onto an amylose column (NEB). After washing the column with 5 column volumes of lysis buffer, ZnJ6 was eluted with 10 mM maltose in the same buffer. Next, the protein was cleaved with TEV protease to remove the MBP tag. The SBP tagged cleaved protein was purified again over a streptavidin–Sepharose (A2S) column. Protein concentration was estimated using a BCA protein assay kit (Thermo Fisher Scientific, Waltham, MA, USA). The SBP-tagged ZnJ6cys > Ser-mutant and MBP proteins were purified similarly. Elution fractions were analyzed on 15% SDS–PAGE (Figure S4).

### 4.4. Subcellular Fractionation of Cytoplasmic and Chloroplast Fractions

Mid-log cells of *Chlamydomonas* (cc-125, 1 L) were harvested and disrupted by nitrogen cavitation in a Yeda press cell disruptor at 100 PSI, which was slowly released. The disrupted cells were centrifuged at 2000× *g* in Corex glass tubes. The supernatant contained the cytoplasmic fraction. The pellets were resuspended in 6 mL of Percoll buffer (330 mM sorbitol, 1 mM MgCl_2_, 20 mM NaCl, 2 mM EDTA, 1 mM MnCl_2_, 2 mM NaNO_3_, 5 mM Na-ascorbate and 50 mM HEPES, pH 7.6). A sample of 5 mL was loaded over a 45/70% Percoll step gradient, which was centrifuged at 20,000× *g* for 10 min at 4 °C in an SW40 rotor. The intact chloroplasts were collected from the interphase of the step gradient. The efficiency of the chloroplast isolation was determined by measuring the chlorophyll content of the purified fraction.

The subcellular fractions were verified by Western blot analysis using gels loaded with equal amounts of protein, the protein concentration of each fraction was estimated using the bicinchoninic acid (BCA) protein assay kit (Thermo Fisher Scientific, Waltham, MA, USA) and the ponceau stains of the Western blots were analyzed using the ImageJ software, to confirm the equal protein loads in the different fractions. Antibodies against organelle-specific proteins were used to verify the subcellular fractions [15]. The RuBisCO small subunit (rbcS, previously raised in our lab [43]) and the 33-kDa oxygen-evolving enzyme (OEE33, obtained from Prof. Zach Adam from the Hebrew University) as chloroplast markers, HSP70A as a cytoplasmic marker obtained from the lab of Prof. Rick Morimoto from Northwestern University. Antibodies against the 32 kDa psbA (Agrisera, Vännäs, Sweden), which encodes the D1 protein of photosystem II served as a marker for the chloroplast and its thylakoids. Antibodies against RA were from Agrisera, Vännäs, Sweden. Antibodies against the recombinant ZnJ6 fragment were generated against amino acids 1–165 (AdarBiotech, Rehovot, Israel), and the interaction is described in Figure S1.

### 4.5. Separation of the Membrane and Soluble Protein Fractions

*Chlamydomonas* cells (cc-125, 250 mL) were grown, pelleted as above, and resuspended in 10 mL, 25 mM HEPES–KOH, pH 7.5, 5 mM MgCl_2_, 0.3 M (10.2%) sucrose with PI. The resuspended pellet was then disrupted with the Yeda Press apparatus at 500 PSI. Membrane and soluble fractions were separated by centrifugation at 100,000× *g* for 1 h at 4 °C. The soluble proteins were precipitated using 20% TCA (final concentration) for 1 h at 4 °C and washed twice with 100% acetone. Pellets of both soluble and membrane proteins were dissolved in 40 mM Tris-HCl pH 7.4, 5 mM EDTA, 4% SDS [44].

### 4.6. Isolation of Thylakoid Membranes

Thylakoid membranes were isolated by a three-step sucrose gradient. 250 mL culture (cc-125) of mid-log cells were grown and harvested as described above. Cells were pelleted at 4500 g for 10 min at 4 °C and resuspended in 25 mM HEPES–KOH, pH 7.5, 5 mM MgCl_2_, and 0.3 M sucrose supplemented with a cocktail of PIs. Cells were disrupted using nitrogen cavitation in the Yeda press cell disruptor at 500 PSI and centrifuged at 2316× *g* (10 min, 4 °C) to separate between the membrane and soluble fractions. The pellet was resuspended in 5 mM HEPES–KOH, pH 7.5, 10 mM EDTA, 0.3 M sucrose, and a cocktail of PIs, followed by centrifugation at 68,600× *g* for 20 min at 4 °C. The resulting pellet was resuspended in 5 mL 5 mM HEPES–KOH, pH 7.5, 10 mM EDTA, 1.8 M sucrose, and a mix of protease inhibitors. The resuspended sample (at the bottom of the tube) was carefully overlaid with 2 mL 5 mM HEPES–KOH, pH 7.5, 1.3 M sucrose, 10 mM EDTA, and then 5 mL, 5 mM HEPES–KOH, pH 7.5, 0.5 M sucrose. The thylakoid membranes were then centrifuged at 247,605× *g* for 1 h at 4 °C. The thylakoid membranes were collected from the interface between the fractions containing 1.8 M and 1.3 M sucrose in the above-mentioned gradient. The thylakoid membranes were spun down at 68,600× *g* for 20 min at 4 °C and washed twice with buffer containing 5 mM HEPES–KOH, pH 7.5, 10 mM EDTA, and a cocktail of PIs. The thylakoid pellet was resuspended in 200 µL of the same buffer [44].

### 4.7. Circular Dichroism and Melting Curves

Circular dichroism measurements were done using a spectropolarimeter (JASCO J-815, Easton, MD, USA) with a 1 mm optical pass cuvette (Hellma, Mullhheim, Germany). The purified recombinant proteins (100 µL) were at a concentration ≥ of 100 µg/mL in Tris buffer, pH 7.5, and loaded into the clean cuvette. Single accumulation spectra ranging between 200 and 260 nm were recorded at RT. Scanning speed was set to 5 nm/min, with 6 sec response time and 1 nm bandwidth. Buffer blank (20 mM Tris, 10 mM NaCl, pH 7.5) without protein served as a control for the experiment. Spectra were baseline corrected by subtracting a blank spectrum.

Melting curves were monitored using the same conditions and buffer. The single wavelength melting curve was generated at a constant wavelength of 222 nm with a temperature range (20–80 °C). The CD Tool software was used to produce principal component analyses (PCA) for each sample. The two main components in the PCA analyses corresponded to spectra of folded and unfolded structures, and their magnitudes were plotted as a function of temperature, providing an overall indication of the thermal stability of the protein.

### 4.8. The PAR-PCMB Zn-Binding Assay

Zn-binding by ZnJ6 was determined using the PAR-PCMB assay, except that the thiol bound zinc was released with para-chloromercuribenzoic acid (PCMB) [17]. Zinc release was measured by its interaction with 4-(2-pyridylazo) resorcinol (PAR) at 500 nm and compared to a ZnCl_2_ standard curve. Metal-free buffers were used throughout the assay following treatment with Chelex 100 resin (5 gr in 40 mM KH_2_PO_4_, pH 7.5) for 1 h at 37 °C. ZnJ6 (3 μM) was mixed with 0.1 mM PAR in 40 mM KH_2_PO_4_ buffer to measure any free or loosely bound zinc in the solution. The addition of 30 μM PCMB to the protein solution (1 mL) caused immediate zinc release and allowed the determination and calculation of the total amount of bound zinc per ZnJ6 molecule. PAR in buffer KH_2_PO_4_ was used as a blank.

### 4.9. Citrate Synthase Assay

The ability to prevent aggregation of heat-sensitive proteins was tested using the citrate synthase (CS) assay, which monitors the holding activity of potential chaperones. ZnJ6 was added in increasing molar ratios (CS: ZnJ6, 1:0.1, 1:1, 1:2, 1:5, 1:10) to CS from porcine heart (Sigma-Aldrich, Rehovot, Israel) in 50 mM Tris pH 8.0 and 2 mM EDTA. CS, 12 μM was denatured by exposure to thermal stress (42 °C) in a 96-well plate containing 200 µL reaction volume in each well. The activity of ZnJ6 was measured by monitoring OD_360_ for 1 h in a plate reader (BioTek Instruments, Winooski, VT, USA). A similar experiment was done using RA as a substrate (Method S3).

### 4.10. Insulin (β-Chain) Aggregation Assay

The thiol-dependent activity of ZnJ6 was examined using the insulin turbidity assay [21]. ZnJ6 and its ZnJ6cys > Ser-mutant were added to insulin in increasing molar ratios (ZnJ6: insulin, 0.2:1, 0.5:1, 1:1) in a solution of 32 μM bovine insulin (Sigma-Aldrich, Rehovot, Israel) (diluted from a stock of 1.7 mM) in a freshly prepared buffer containing 0.1 M potassium phosphate (pH 7.0) and 2 mM EDTA (100 µL). The reaction was initiated by the addition of freshly prepared DTT to a final concentration of 1 mM at 25 °C. A reaction mix containing insulin alone served as control. Precipitation of the insulin β-chain was measured at 650 nm for 2 h in a 96-well plate.

### 4.11. Ellman’s Test for Determination of Protein-Bound Sulfhydryl (PB-SH) Groups

To calculate the sulfhydryl (-SH) group bound to a protein in the reaction mix, ZnJ6 was added to bovine insulin in an equimolar ratio; ZnJ6 alone served as control. One mM DTT was added to the solution to reduce the insulin, as described above. The amount of protein bound-SH (PB-SH) in the reaction mix was calculated before and after incubation for 2 h. To calculate the PB-SH, DTNB was first added to the mixture, and total -SH groups (T-SH) were measured by taking absorbance at 412 nm [before precipitation with trichloroacetic acid (TCA)]. Next, a parallel mixture was TCA precipitated, centrifuged to remove all the proteins, and the DTNB was added to the protein-free supernatant. This step eliminated the effect of DTT on binding to DTNB. The value obtained was nonprotein bound-SH (NP-SH). The difference between the T-SH and the NP-SH values gave the PB-SH groups [45]. Total-SH groups were quantified by adding 50 µL of reaction sample in 950 µL DTNB reagent (0.1 mM DTNB, 2.5 mM sodium acetate, and 100 mM Tris, pH 8). For each measurement of DTNB binding before and after TCA precipitation was measured. The mixture was incubated for 5 min at RT before monitoring the absorbance at 412 nm.

### 4.12. Reduced and Denatured RNaseA Reactivation Assay

Reduced and denatured RNaseA (rdRNaseA) was prepared by overnight incubation of the native RNaseA enzyme (Sigma-Aldrich, Rehovot, Israel) (20 mg/mL) in 500 μL of 0.1 M Tris-HCl pH 8.6 containing 150 mM DTT and 6 M GnHCl. Excess DTT and GnHCl were separated from the rdRNaseA using a Sephadex G-25 buffer replacement column equilibrated with 10 mM HCl. RNaseA aliquots (10 mg/mL stock) were stored at −80 °C. Reactivation of rdRNaseA was initiated by 200-fold dilution of the protein (to a final concentration of 50 μg/mL (3.8 μM) in 1 mL of reactivation buffer (0.1 M Tris-HCl pH 7.0, 0.1 M NaCl and 1 mM EDTA). The reactivation was performed in the absence or presence of increasing molar ratios (ZnJ6:rdRNaseA, 0.2:1, 0.6:1, and 1.2:1). Aliquots (50 μL) were removed at various intervals and mixed with 50 μL assay mix containing 0.1 M Tris- HCl pH 7.2, 0.1 M NaCl, and 0.3 mg/mL cytidine 2′,3′-cyclic monophosphate. RNaseA activity was measured by monitoring the hydrolysis of cytidine 2′,3′-cyclic monophosphate at 284 nm. The hydrolysis was calculated as the difference between OD_284_ at t = 0 min and t = 10 min. Reactivation was presented as a percentage of hydrolysis of treated samples compared to the hydrolysis of native RNaseA [9].

### 4.13. Analysis of Proteins that Associate with ZnJ6 by Pull-Down Experiments

Recombinant ZnJ6 fused to a 6 kDa streptavidin-binding peptide (SBP) tag (100 μL, 10 μM) was incubated with streptavidin–Sepharose resin (A2S). Chloroplasts (4 mL, 0.1 μg/μL) were isolated and solubilized on ice for 5 min using 1% β-DDM (n-dodecyl β-D-maltoside, Sigma-Aldrich, Rehovot, Israel) in a buffer containing 0.7 M sucrose, 0.1 M Tris-HCl, 0.3 M NaCl, pH 7.5 and a cocktail of PIs (Sigma-Aldrich, Rehovot, Israel). The sample was centrifuged for 40 min at 40,000× *g*, and the soluble protein fraction was collected, diluted 1:10 in the buffer (20 mM Tris-HCl, pH 7.4, 200 mM NaCl, 1 mM EDTA), and loaded onto the streptavidin–Sepharose beads (200 μL) following their incubation with the recombinant the ZnJ6 protein. The mixture was incubated at 4 °C for 2 h. The beads were then washed with 5 column volumes of the buffer (pH 7.4) to remove nonspecific proteins. Finally, the bound protein with its associated complex was eluted using 2 mM biotin. SBP-tagged MBP treated similarly served as a control for nonspecific binding of proteins to the beads.

### 4.14. Mass Spectrometry (MS)

The gel lane containing the proteins that were co-eluted from the streptavidin–Sepharose column were extracted from the gel and further reduced using 3 mM DTT (60 °C for 30 min), followed by modification with 10 mM iodoacetamide in 100 mM ammonium bicarbonate for 30 min at 25 °C. The sample was subsequently treated with trypsin (Promega, Madison, WI, USA) and digested overnight at 37 °C in 10 mM ammonium bicarbonate. Digested peptides were desalted, dried, resuspended in formic acid (0.1%) and resolved by reverse phase chromatography over a 30 min linear gradient with 5% to 35% acetonitrile and 0.1% formic acid in the water, a 15 min gradient with 35% to 95% acetonitrile and 0.1% formic acid in water and a 15 min gradient at 95% acetonitrile and 0.1% formic acid in water at a flow rate of 0.15 µL/min. Mass spectrometry was performed using Q-Exactive Plus mass spectrometer (Thermo Fisher Scientific, Whitbym, ON, Canada) in the positive mode set to conduct a repetitively full MS scan along with high-energy collision dissociation of the 10 dominant ions selected from the first MS scan. A mass tolerance of 10 ppm for precursor masses and 20 ppm for fragment ions was set. All analyses were performed in triplicate. The MS analyses were performed at the Smoler Center of the Technion Institute of Technology.

### 4.15. Statistical Analysis

Raw mass spectrometric data were analyzed using MaxQuant software version 1.5.2.8. The data were searched against *C. reinhardtii* proteins listed in the Phytozome database. Protein identification was set at less than a 1% false discovery rate. The MaxQuant settings selected were a minimum of 1 razor/unique peptide for identification, a minimum peptide length of six amino acids, and a maximum of two mis-cleavages. For protein quantification, summed peptide intensities were used. Missing intensities from the analyses were substituted with values close to baseline only if the values were present in the corresponding analyzed sample. LFQ intensities were compared between the three SBP-ZnJ6 biological repeats and the three SBP-MBP repeats on the Perseus software platform using a Student’s *t*-test.

The enrichment threshold (LFQ intensity of SBP-ZnJ6 subtracted from SBP-MBP control) was set to a log2-fold change ≤ −3 (8-fold enrichment compared to control) and *p*-value < 0.05. The filtered proteins were categorized both manually, based on their function in the Phytozome database, and using BLAST2GO software based on the biological process (with a minimum of 2-fold enrichment) as selected criteria. The minor categories (sub-branches) with the BLAST2GO software were merged into four broader classes: photosynthesis, metabolic process, oxidoreductases, and transporters.

### 4.16. Redox Sensitivity of Chlamydomonas Cells by Exposure to H_2_O_2_, MeV, or DTT

To verify the in vivo function of ZnJ6, the insertional knock-down mutant ΔZnJ6, with the paromomycin resistance, was used (LMJ.RY0402.048147). *C. reinhardtii* cells (ΔZnJ6 and the WT background strain cc-4533) were grown to mid-log phase in HS medium with a light intensity of 150 µmol/s/m^2^. The mutant was confirmed by PCR and western analysis using anti-ZnJ6 antibodies (Figure S8). For all treatments, ~1 × 10^7^ cells mL^−1^ (1 mL) were exposed to different concentrations of H_2_O_2_ (2, 5, 10, and 20 mM), MeV (2, 5, 10, and 20 µM), and DTT (2, 5, 10 and 20 mM). After exposure of 2 h, the cells were washed twice and resuspended in HS media with required dilution. A total of 10^3^ cells (3 µL) were seeded over HS plates and allowed to grow at 23 °C. Pictures were taken on day 5 of the growth and analyzed using MultiGauge software. Paired *t*-test was performed on at least 3 replicates to compare the response of ΔZnJ6 and the WT background strain to different treatments.

### 4.17. Exposure of Chlamydomonas Cells to Heat Stress

*C. reinhardtii* cells (ΔZnJ6, WT background cells) as described above. 5 µL (serially diluted from 10^5^ to 10^2^ cells/5 µL) aliquots were spotted on HS plates and incubated at 23 °C (control) or exposed to heat stress by incubating the plates at 37 °C for 1, 2, and 4 h. Pictures were taken on day 5 of the growth, and the density was measured using MultiGauge software followed by *t*-test analysis.

### 4.18. Analysis of Chlorophyll Fluorescence Transient/Fast OJIP Analysis

Fluorescence of Chlorophyll a was taken using AquaPen-C AP 110-C. Mid-log *Chlamydomonas* cells (2 mL) with a constant chlorophyll concentration of 2 mg/L for each sample (WT cc-4533, the ∆ZnJ6, with or without H_2_O_2_ treatment). Measurements were taken after turning off the actinic light for at least 5 min prior to the measurements. The fast chlorophyll fluorescence induction kinetics were done with saturating flash intensity of ca. 3000 µmol m^−2^ s^−1,^ and measurement time was 1 sec. The plotted curve (Figure 10a,b) is a mean of three independent repeats. In order to compare the F0/Fm and mid-log transition, the curve was normalized to Fm and relative variable fluorescence (Vt) curves (Figure 10c,d). The Vt curve was obtained by Vt = (Ft − F0)/(Fm − F0).

## Data Availability

Not applicable.

## Supplementary Materials

Supplementary Materials can be found at https://www.mdpi.com/1422-0067/22/3/1136/s1. Figure S1: Generation of anti-ZnJ6 antibodies, Figure S2: Prediction of a transmembrane domain in ZnJ6, Figure S3: ZnJ6 is a putative chloroplast protein, Figure S4: Purified recombinant proteins separated over 12% SDS-PAGE, Figure S5: Recombinant Rubisco Activase (RA) purification, Figure S6: ZnJ6 prevents the aggregation of RA at elevated temperatures, Figure S7: Chloroplast isolation from *Chlamydomonas* cells and pull-down assay, Figure S8: Characterization and verification of the CLiP mutant LMJ.RY0402.048147, Table S1: Mass spectrometry analysis of pulled down proteins, Table S2: Primers for recombinant protein expression, Table S3: Primers for Cysteine to Serine (TGC > TCC) mutagenesis in ZnJ6, Method S1: Cloning for recombinant protein expression, Method S2: Cloning and purification of recombinant RA, Method S3: Prevention of aggregation of RA by ZnJ6 in vitro.

## Abbreviations

Cys	Cysteine
CS	Synthase
DTT	Dithiothreitol
MeV	Methyl viologen
PS I	Photosystem I
PS II	Photosystem II
rdRNase A	Reduced denatured RNase A
SBP	Streptavidin-binding peptide
Zn	Zinc
ZF	Zinc finger
