# Supplementary material for: ZnJ6 Is a Thylakoid Membrane DnaJ-Like Chaperone with Oxidizing Activity in Chlamydomonas reinhardtii
Source: Int J Mol Sci. 2021 Jan 24;22(3):1136. doi: 10.3390/ijms22031136 (PMC7865324; doi:10.3390/ijms22031136)
Supplement: Supplementary file 1 [file ijms-22-01136-s001.zip › Supplementary Material/Supplementary Material.pdf]

Supplementary Materials

Supplementary Tables

Table S2. Primers for recombinant protein expression

C1	Bam HI-ZnJ6 (CTP) Fw	GGATCCATGGCTTCACCGTTGCTTGC
C2	XhoI (No Stop) ZnJ6 Rev	CTCGAGATCCAGCGGCCGTGATGACAC
R1	Nco1 SBP Fw	GCCATGGATATGGACGAGAAGACCAC
R2	EcoR1 SBP Rev	CAGAATTCGGGCTCGCGCTGGCCCTGAG
R3	Bam H1 ZnJ6 (756 bp) Fw	GGATCCATGCGCAGAAGTGCCACTGAC
R4	Bam H1 ZnJ6 Rev	GGATCCATCCAGCGGCCGTGATG
R5	EcoR1 GB1 Fw	CAGAATTCATGCAGTACAAACTGATCC
R6	Xho1 ZnJ6 (STOP) Rev	CTCGAGCTAATCCAGCGGCCGTGATG
R7	Nco1 ZnJ6 (756 bp) Fw	CCATGGATGCGCAGAAGTGCCACTGAC
R8	Xho1 ZnJ6 (489 bp)(Stop) Rev	CTCGAGTTAGGACTTTACTTTGCGCACCAG
R9	Eco R1 Rubisco activase Fw	GACCAAGAATTCATGCAGGTCACCATGAAGAG
R10	Xho1 Rubisco activase Rev	CTGAGTCTCGAGTTAGCGGCTGTAGGCCTC

Table S3. Primers for Cysteine to Serine (TGC > TCC) mutagenesis in ZnJ6

M1	Fw (G>C, 491 & 500)	GCAAAGTAAAGTCCTCCCGCTGCTCCAAGGGCTTCGGCGT
M2	Rev (G>C, 491 & 500)	ACGCCGAAGCCCTTGGAGCAGCGGGAGGACTTTACTTTGC
M3	Fw (G>C, 524 & 533)	TCGGCGTGGTGCGCTCCCGGCTGTCCGACGGCCGCGGCAC
M4	Rev (G>C, 524 & 533)	GTGCCGCGGCCGTCGGACAGCCGGGAGCGCACCACGCCGA
M5	Fw (G>C, 587 & 596)	CCTACTCGGAGACCTCCCCGCTCTCCGCAGCCAAGCGCTT
M6	Rev (G>C, 587 & 596)	AAGCGCTTGGCTGCGGAGAGCGGGGAGGTCTCCGAGTAG
M7	Fw (G>C, 620 & 629)	AGCGCTTCGTGGTGTCCCCCGACTCCGGCGGCCACTACCA
M8	Rev (G>C, 620 & 629)	TGGTAGTGGCCGCCGGAGTCGGGGGACACCACGAAGCGCT

Supplementary Methods

Method S1

Cloning for recombinant protein expression

Early log-phase cells (OD_{750} = 0.25-0.35, 2×10^6 cells/ml) were used to isolate total RNA by the TRI Reagent protocol (Sigma). The cDNA was synthesized using the High Capacity cDNA reverse transcription protocol (Applied Biosystems) with 1 µg RNA as a template.

ZnJ6 (full length along with CTP, 870 bp) from *C. reinhardtii* was PCR amplified from cDNA using primers C1/C2 (Table S2) and cloned first into the pJET1.2/blunt plasmid (Plasmid 1) using the Clone JET PCR cloning kit (Thermo Scientific). The primer pair R3/R4 was used to PCR amplify the full-length ZnJ6 (756 bp, without the CTP), using plasmid 1 as template. The PCR fragment was further cloned in the pET30-GBFusion1 (Plasmid 2) expression vector downstream of the T7 promoter. GB1 is a 6 kDa solubility-inducing domain [1]. To make the final clone for recombinant protein expression a SBP tag was added downstream of the N-terminal cleavable maltose-binding protein (MBP)-tag in the pMBP-Parallel2 vector [2]. The SBP tag was amplified using the primer pair R1/R2 from a pJET-SBP plasmid and ligated into the pMBP-Parallel2 vector. Using the primer set R5/R6, the GB1-ZnJ6 open reading frame was amplified from plasmid 2 and cloned downstream of the SBP tag in the pMBP-Parallel2 vector (Plasmid 3). A ZnJ6 mutant was generated, ZnJ6cys>ser, in which all cysteine residues of the ZF domains were substituted for serines. This exchanged cys residues 164, 167, 218, 221 for the first Zn binding motif, and positions 175, 178, 207 and 210 for the second, using site-directed mutagenesis. Plasmid 3 was used as a starting template for the mutagenesis, and the primers (M1-8, Table S3) were designed to exchange the cys residues in the protein. Each mutation was confirmed by DNA sequencing, and the final mutant version was Plasmid 4.

Generating Antibodies against ZnJ6

Since *Chlamydomonas* has many ZF proteins, non-specific reactions with antibodies raised against a protein that contains this domain could be expected. Therefore, to improve the antibody specificity, a shorter version of the recombinant protein ZnJ6 (114-609 bp) corresponding to amino acids 38-203, without the N-terminal CTP and the zinc finger domain (Plasmid 5) was expressed in *E. coli* (Rosetta cells). Due to solubility issues, the ZnJ6 fragment was first expressed as a fusion protein with MBP in the pMBP parallel2 vector. The recombinant protein was purified over an

amylose column, and the MBP tag was then removed from the purified protein by cleavage with the TEV protease.

Method S2

Cloning and purification of recombinant Rubisco activase (RA)

Full length RA (1227 bp) was amplified from cDNA using the R9/R10 primers (Table S2). The PCR product was cloned into the blunt end pJET1.2 cloning vector. RA was amplified from the plasmid and cloned downstream of the SBP-tag that was introduced into the pMBP-Parallel2 vector (Plasmid 3), using *Eco*RI and *Xho*I restriction sites. Bacterial BL21 cells were transformed with the expression plasmid. Recombinant protein expression and purification were as for recombinant ZnJ6 protein with slight modifications. The MBP and SBP tagged RA was cleaved using TEV to remove the MBP tag. SBP tagged cleaved protein was repurified over a streptavidin column.

Method S3

Prevention of aggregation of Rubisco activase by ZnJ6 in *vitro*

Rubisco activase is a temperature-sensitive protein. We checked whether ZnJ6 could also prevent RA aggregation in *vitro* at an elevated temperature (42 ºC) as it does for CS. We added ZnJ6 to RA in a 3:1 molar ratio in a buffer containing 50 mM Tris pH 8.0 and 2 mM EDTA. The activity of ZnJ6 was measured by its ability to prevent the aggregation of RA. The effect of ZnJ6 on the prevention of RA aggregation was measured by monitoring OD_{360} for an hour**.** An increase in aggregation resulted in an increase in absorbance. ZnJ6 alone in the buffer served as a control. Buffer alone was used as blank.

Supplementary Figures

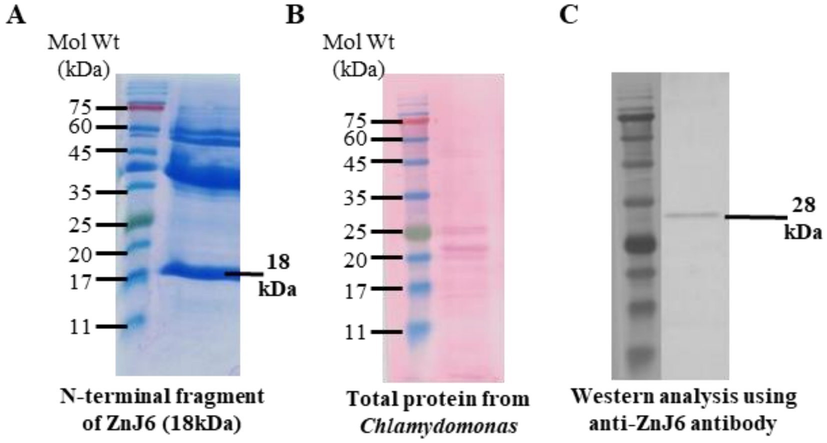

Figure S1. Generation of anti-ZnJ6 antibodies. A. The ZnJ6 fragment corresponding to amino acids 38-203 was cleaved from plasmid 5 by TEV. The resulting polypeptide was separated on 12% SDS PAGE, and the specific band (18 kDa) was extracted and used to raise antibodies in New Zealand rabbits (Adar Biotech, Israel). **B**. A ponceau stain of blotted total *Chlamydomonas* proteins. **C.** Western analysis using the anti-ZnJ6 antibody (dilution, 1:500). The band of expected size (28 kDa) of ZnJ6 is marked.

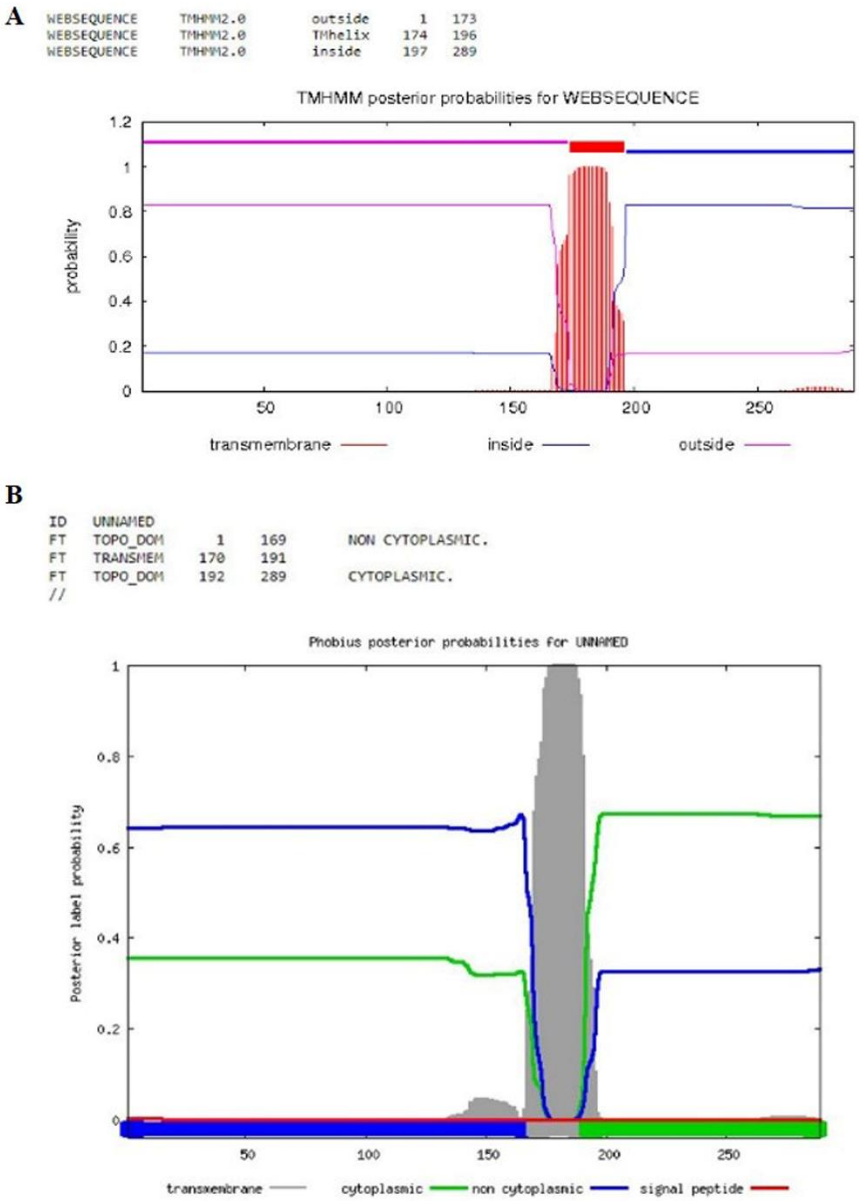

Figure S2. Prediction of a transmembrane domain in ZnJ6. ZnJ6 sequence was applied to the TMHMM (A) and Phobius (B) online transmembrane prediction servers to search for a predicted transmembrane domain. The predicted transmembrane region is shown in red and grey in the two respective servers.

A

```
### targetp v1.1 prediction results ##################################
Number of query sequences:  1
Cleavage site predictions not included.
Using PLANT networks.

Name                   Len     cTP    mTP     SP  other  Loc  RC
----------------------------------------------------------------------
Sequence               870   0.249  0.079  0.051  0.559   _     4
----------------------------------------------------------------------
cutoff                       0.000  0.000  0.000  0.000
```

B

```
### chlorop v1.1 prediction results ############################
Number of query sequences: 1

Name                     Length     Score  cTP       CS-    cTP-
                                                   score  length
-----------------------------------------------------------------
Sequence                    289     0.548   Y      5.330      51
-----------------------------------------------------------------
```

Figure S3. ZnJ6 is a putative chloroplast protein. Prediction was done using online servers, TargetP and ChloroP. **A.** TargetP gives a high score for cTP (chloroplast transit peptide) than for mTP (mitochondrial transit peptide) and SP (secretory pathway signal peptide), indicating the localization of ZnJ6 in the chloroplast. **B.** ZnJ6 protein sequence was also applied to the ChloroP bioinformatics server. Y in the cTP column accounts for the presence of the chloroplast target sequence.

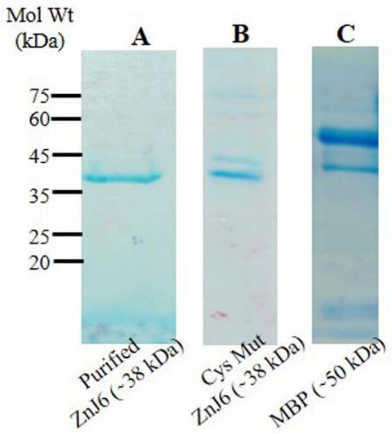

Figure S4. **Purified recombinant proteins separated over 12% SDS-PAGE. A**. Purified SBP-tagged ZnJ6 (~38 kDa). **B**. Purified SBP-tagged ZnJ6cys>ser mutant (~38 kDa), where all the cysteines of the zinc-finger domain were replaced with serines. **C.** Purified SBP-tagged MBP of~50 kDa used as control in the experiments. The 43 kDa in the gel is MBP without the SBP tag.

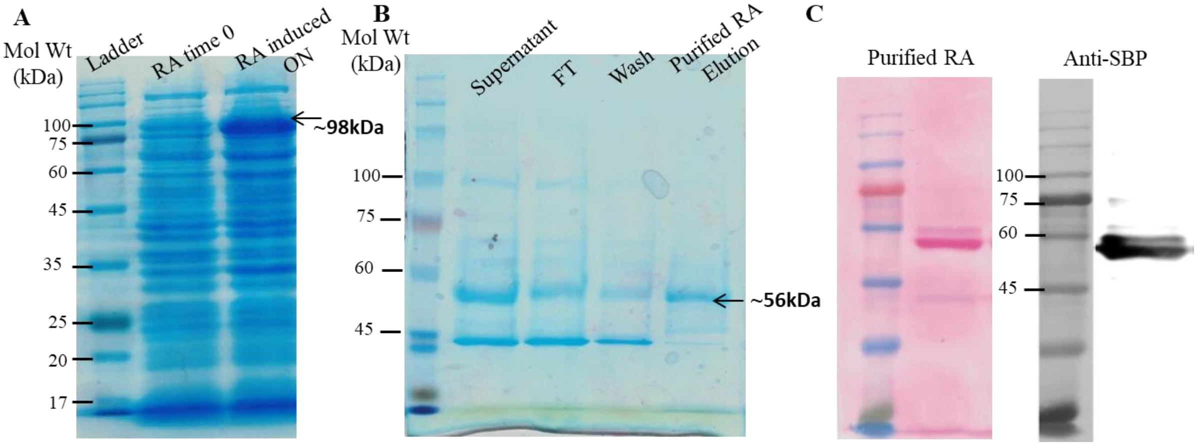

Figure S5. Recombinant Rubisco activase (RA) purification. **A**. Rubisco activase was sequentially tagged with MBP and SBP at its N-terminus (MBP-SBP-RA, 98 kDa). A sample of uninduced total proteins from *E.coli* at time zero and a sample from induced expression performed overnight at 20 °C in the presence of 0.5 mM IPTG. **B**. Purification of the SBP-tagged RA over a streptavidin affinity column after cleaving off the MBP tag with the TEV protease. The supernatant, flow through (FT) and washes contained the cleaved MBP (43kDa) and the 56 kDa SBP-tagged RA. The elution fraction contained the purified recombinant SBP-tagged RA. **C.** Purified RA was verified using anti-SBP antibodies.

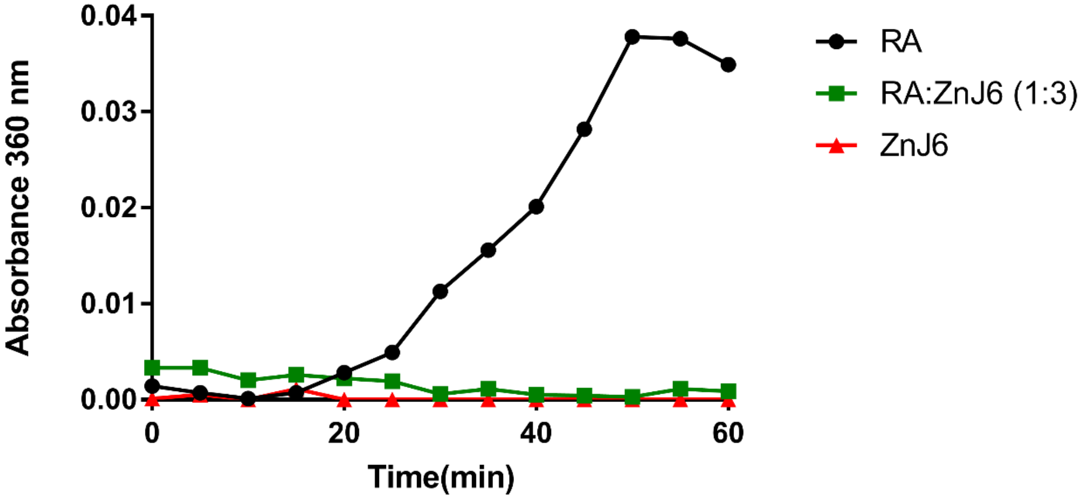

Figure S6. ZnJ6 prevents the aggregation of Rubisco activase (RA) at elevated temperatures. Ability of ZnJ6 to prevent thermal aggregation of temperature sensitive RA was measured by adding ZnJ6 at a 1:3 molar ratio (RA: ZnJ6). The absorbance was measured at 360 nm, for 1 hr at 42 ºC. The increase in aggregation corresponds to an increase in absorbance.

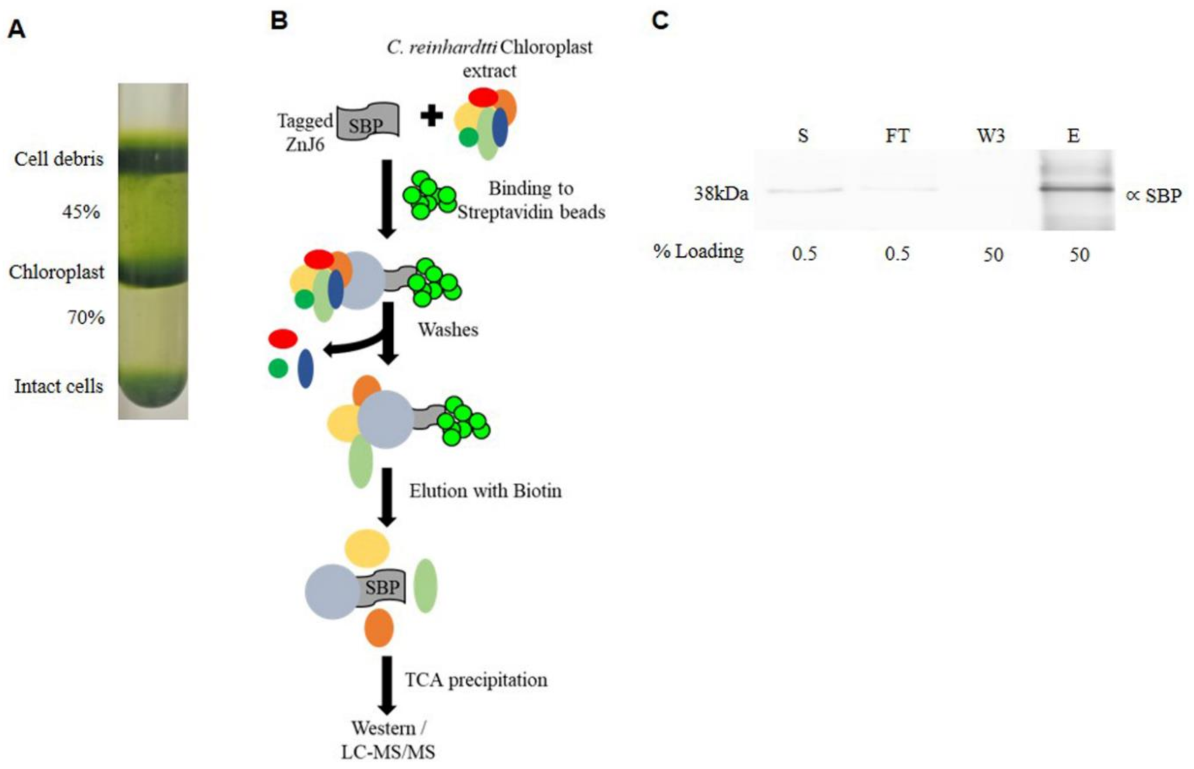

Figure S7. Chloroplast isolation from *Chlamydomonas* cells and pull-down assay. A. The two step gradient of Percoll for chloroplast isolation. The intact chloroplasts were collected from the interphase between the 45 and 70 % fractions of the Percoll gradient. **B**. Schematic representation of the pull-down assay using recombinant SBP tagged ZnJ6 protein first bound to streptavidin-Sepharose and then incubated with the *Chlamydomonas* chloroplast extract for 2 hours. The beads were then washed to remove the non-specific proteins until reaching a protein-free wash fraction. Finally, the recombinant ZnJ6-SBP bait protein was eluted with biotin, along with its interacting proteins. Recombinant SBP-tagged MBP was used as an experimental control that was treated similarly. The eluted fractions were analyzed by MS. **C.** Western analysis of different samples from the pull- down assay. Supernatant (S), flow through (FT), final wash (W3) and elution (E).

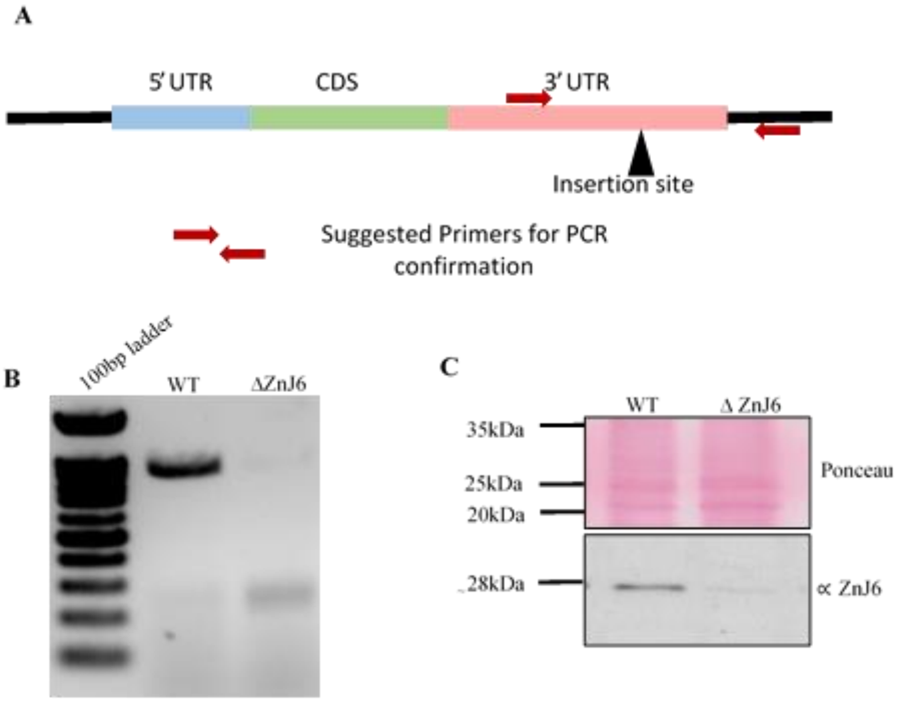

Figure S8. Characterization and verification of the CLiP mutant LMJ.RY0402.048147. **A**. Diagramatic representation of the insertion site for the CLiP mutant. **B.** Colony PCR to confirm the insertion site using suggested primers (**1**. TCCAGTCAGTCAGTTGGCAG **2**. TGCAGTTGTACGGCTTTTTG). Wild type, background strain cc-4533 shows amplification, whereas in the ZnJ6 mutant there is no amplification, as the site is disrupted **C.** Western analysis using anti-ZnJ6 antibodies shows reduced expression of protein in ΔZnJ6.

References

1. Cheng, Y.; Patel, D.J. An efficient system for small protein expression and refolding. *Biochem. Biophys. Res. Commun.* **2004,** *317(2)*, 401-405.

2. Sheffield, P.; Garrard, S.; Derewenda, Z. Overcoming expression and purification problems of RhoGDI using a family of "parallel" expression vectors. *Protein Expr. Purif.* **1999**, *15*, 34–39.
